# Anionic Polysaccharides: Promising 3D Bioink Candidates for Tissue Engineering

**DOI:** 10.3390/bioengineering13040408

**Published:** 2026-03-31

**Authors:** Feiyang Wang, Redouan El Boutachfaiti, Gustavo Cabrera-Barjas, Cédric Delattre

**Affiliations:** 1 Université Clermont Auvergne, Clermont Auvergne INP, Institut Pascal, Centre National De La Recherche Scientifique (CNRS), 63000 Clermont-Ferrand, France; feiyang.wang@doctorant.uca.fr; 2 UMRT INRAE 1158 BioEcoAgro, BIOlogie des Plantes et Innovation (BIOPI), Avenue des Facultés, IUT d’Amiens, Université de Picardie Jules Verne, Le Bailly, 80025 Amiens, France; redouan.elboutachfaiti@u-picardie.fr; 3 Facultad de Ciencias de la Rehabilitación y Calidad de Vida, Escuela de Nutrición y Dietética, Universidad San Sebastián Campus Las Tres Pascualas, Lientur 1439, Concepción 4080871, Chile; gustavo.cabrera@uss.cl; 4 Institut Universitaire de France (IUF), 1 rue Descartes, 75005 Paris, France

**Keywords:** anionic polysaccharide, tissue engineering, 3D bioprinting, synthetic polysaccharide

## Abstract

Anionic polysaccharides are sugars with a negative charge. Common examples include alginic acid, hyaluronic acid, pectin, chondroitin sulfate, and heparin. These molecules are bioactive and widely used in medicine, food, and cosmetics. On their own, they usually do not bind to cells. However, they can serve as bioinks or as parts of bioink mixtures. In 3D bioprinting, anionic polysaccharides are often combined with natural polymers like collagen or with synthetic materials to form hydrogels. These hydrogels act as scaffolds that give cells a three-dimensional space to grow and form tissue. The properties of these hydrogels can be tuned to match specific needs. Together, anionic polysaccharides and 3D bioprinting offer strong potential for tissue engineering, making it possible to build complex, custom tissue structures that may help solve problems in organ repair and replacement. This review summarizes the sources, structures, and key properties of Common anionic polysaccharides, with a comparative analysis of their chemical, biological, and mechanical characteristics and recent 3D printing strategies. It highlights the advantages of anionic polysaccharide bioinks, particularly their compatibility with enzymatic, photo-, and ionic crosslinking, and presents representative examples demonstrating their suitability for diverse tissue engineering applications and printing requirements.

## 1. Introduction

Polysaccharides are made of two or more simple sugars. Their physical properties depend mainly on how the sugar units are arranged and linked together. They are often used in 3D bioprinting hydrogels because they are stable, biodegradable, safe, and easy to modify [1]. Based on their charge, polysaccharides can be grouped into three types: (1) neutral ones such as amylose, guar gum and cellulose; (2) cationic ones such as chitosan; and (3) anionic ones such as alginate, pectin, heparin, and xanthan gum. Natural anionic polysaccharides are mostly found in the extracellular matrix of animal tissues and in marine algae. They are usually present as sulfated glycosaminoglycans (GAGs) [2].

Natural polysaccharides take part in many biological processes. They are linked to cell recognition, cell growth, metabolism, cancer treatment, anti-inflammatory responses, blood clot prevention, antibacterial activity, antioxidant effects, antitumor activity, and immune regulation [3,4]. Anionic polysaccharides can be modified through their carboxyl and hydroxyl groups. This increases their range of uses in tissue engineering. After chemical changes, they can be turned into many useful forms. These include nanogels, electrospun scaffolds, porous scaffolds, and injectable hydrogels [5]. However, natural anionic polysaccharides face several challenges when used in tissue engineering. They can break down too quickly, take up too much water, dissolve poorly, and vary in molecular weight depending on their source. They can also be prone to microbial contamination. For these reasons, synthetic anionic polysaccharides are gaining attention. By using grafting and crosslinking, researchers can take advantage of the many hydroxyl and carboxyl groups in these polymers. Such modifications help control degradation, solubility, and flow properties, making them better suited for industrial use [6]. Techniques such as TEMPO-mediated oxidation, carboxymethylation, phosphorylation, sulfation, and selective etherification or esterification reactions are widely employed to introduce anionic functional groups (e.g., carboxylate, phosphate, or sulfate moieties) onto polysaccharide backbones, thereby converting native or weakly charged polysaccharides into anionic derivatives [7]. These chemical modifications enable effective ionic crosslinking with divalent cations such as Ca^2+^, facilitating the formation of physically crosslinked hydrogel networks. Among these approaches, TEMPO-mediated oxidation is currently the most extensively used method due to its high reaction efficiency, mild aqueous reaction conditions, and remarkable regioselectivity toward the primary C6 hydroxyl groups of polysaccharides, which allows precise control over the degree of oxidation while largely preserving the polymer backbone and biocompatibility.

3D bioprinting is seen as a promising way to address the shortage of organs for transplantation [8]. This technology can achieve unparalleled structural control, adaptability and reproducibility, overcoming the limitations of traditional biomanufacturing technology. Several printing methods exist, including extrusion, inkjet, pressure-assisted, laser-assisted, and stereolithography. Of these, extrusion is the most widely used because it is compatible with many bioinks. Bio-inks are biomaterials that encapsulate living cells. Bioprinting can rapidly construct high-resolution, large-scale structures, giving it great application potential in the field of tissue engineering [9]. The mechanical and biological properties of bio-inks are key elements of bioprinting, determining the stability of the structure and the survival rate of cells. Choosing the right bioink is the most important step [10]. Natural polymer materials that can structurally and functionally mimic the extracellular matrix (ECM) are most readily recognized and accepted by organisms in vivo [11]. Polysaccharides are common choices, as they can form new inks for tissue engineering materials [12]. Their many modifiable groups, especially carboxyl and hydroxyl groups, allow the formation of crosslinked 3D networks with adjustable mechanical properties [13]. The most studied polysaccharides in bioprinting include hyaluronic acid, alginate, chondroitin sulfate, and chitosan [14]. They offer useful features such as good flow properties, biodegradability, non-toxicity, printability, and biocompatibility [15]. Among them, anionic polysaccharides stand out. Their many carboxyl groups make them easy to modify chemically or physically. In this paper, we first present the structures of natural anionic polysaccharides to highlight their functional groups. These functional groups serve as the theoretical basis for synthesizing anionic polysaccharides. Then, we demonstrate the tissue engineering applications of hydrogels prepared by enzymatic, photo-crosslinking, and ionic crosslinking methods, and explain their advantages. We aim to give an overview of how anionic polysaccharides and their derivatives are used in 3D bioprinting for tissue engineering.

We first present the sources, structures, basic properties, and unique properties of anionic polysaccharides. We then provide an in-depth description of the chemical, biological, and mechanical characteristics of each polysaccharide, as well as the latest application strategies and methods in 3D printing. Finally, we demonstrate specific application scenarios using several application examples of anionic polysaccharides.

## 2. Anionic Polysaccharides Description

### 2.1. Natural Anionic Polysaccharides

Anionic polysaccharides are a class of complex carbohydrates that bear negatively charged functional groups along their molecular structures. Polysaccharides are large molecules composed of repeating units of simple sugar molecules (monosaccharides) linked together by glycosidic bonds. Anionic polysaccharides have additional functional groups such as carboxylate (-COO^−^) or sulfate (-SO_3_^−^) attached to their sugar units

#### 2.1.1. Alginate

**Figure 1:**
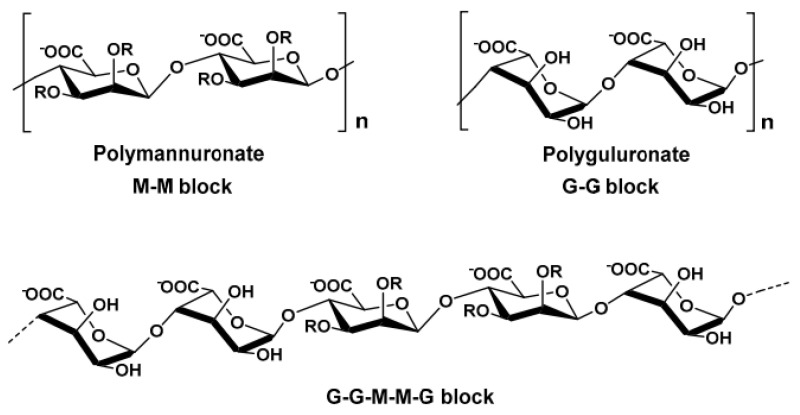
Main structure of alginates. From algae with R=H and from bacteria with R=H or COCH; (acetyl group) [22].

Alginic acid is a naturally occurring anionic polysaccharide that is widely found in nature. It is mainly extracted from brown seaweeds such as kelp and sargassum, but it can also be produced by certain bacteria, including nitrogen-fixing bacteria and Pseudomonas species [16]. Alginate is known for its excellent biocompatibility, non-toxicity, and mild gelation behavior. It can easily crosslink with divalent metal ions such as calcium (Ca^2+^) to form soft hydrogels. Because of these properties, alginate has been widely used in many areas, including biomedicine, food processing, textile printing, and dyeing [17]. Structurally, alginic acid is made up of two types of sugar acids: (1,4)-β-D-mannuronic acid (M) and α-L-guluronic acid (G), as shown in Figure 1. Each sugar ring contains one carboxyl group (-COOH) and two hydroxyl groups (-OH) that face in different directions. These two monomers are connected by 1,4-glycosidic bonds and arranged in three block patterns—MM blocks, GG blocks, and MG blocks—forming a linear, unbranched copolymer. The composition and arrangement of these blocks vary depending on the source of the alginate. Differences in the ratio, distribution, and length of M and G blocks strongly influence the physical and chemical behavior of the polymer, such as its viscosity, gel strength, and elasticity [18]. In addition to its rigid structure, alginic acid offers good biocompatibility, low cost, and mild gel-forming conditions. These features make it useful in many different fields [19]. Sodium alginate (SA) can form soft gels under mild conditions. Its G-unit’s sodium ions (Na^+^) can exchange with divalent or trivalent cations to create firm gels. This gel formation happens when carboxylic acid groups on the SA chain bond with these cations. In fact, SA can combine with various divalent and trivalent metal ions to form gels, but the combination mechanism and gel properties vary depending on the type of ion. Among them, divalent alkaline earth metals, calcium (Ca^2+^), barium (Ba^2+^), and strontium (Sr^2+^) are the most classic class of gelling ions. Calcium ions (Ca^2+^) play a special role in this process. They fit into electronegative pockets, creating what scientists call an “egg carton” structure. These binding zones connect the polymer chains together, turning the liquid solution into a gel [20]. Furthermore, the interactions between Cu^2+^, Ni^2+^, Zn^2+^ and SA molecules are very strong, and they can even form covalent bonds. The order of binding affinity is Cu^2+^ > Ca^2+^ > Co^2+^ > Ni^2+^ > Zn^2+^. Trivalent ions can cross-link with alginate through stronger coordination. Examples include iron (Fe^3+^) and aluminum (Al^3+^).

Alginate’s solubility depends on three main factors: (1) the solvent’s pH level, (2) the medium’s ionic strength, and (3) the presence of gelling ions in the solvent. Because alginate contains many polar groups, it shows strong water-attracting properties. This makes it easily dissolve in water but not in organic solvents. When hydrochloric acid is added, it changes alginate’s carboxyl groups into alginic acid. This new form cannot dissolve in water. However, adding alkali can convert the alginic acid back to soluble alginate [21].

Alginate is widely used in tissue engineering and bioprinting because of its low toxicity, biodegradability, and good biocompatibility. These properties arise from several intrinsic features. Alginate gels formed by ionic crosslinking can be dissolved by monovalent ions, which allows material removal or remodeling. However, alginate lacks native bioactive motifs, resulting in weak cell adhesion [23]. In addition, alginate hydrogels often exhibit limited mechanical strength after swelling in aqueous environments [24]. Alginate also shows low electrical and thermal conductivity and does not possess inherent antibacterial activity. The gelation behavior and mechanical properties of alginate can be tuned by controlling the composition and distribution of guluronic acid (G) and mannuronic acid (M) blocks [25]. Crosslinking of G blocks with Ca^2+^ ions follows the classical “egg-box” model. Alginate with a high G/M ratio (>1.5) forms dense and rigid networks with increased compressive strength. In contrast, alginate with a higher M/G ratio (>1.5) produces softer and more flexible gels, showing higher elongation but reduced stiffness. Several strategies have been explored to improve the printability of alginate-based bioinks. Blending alginates with different molecular weights can enhance flow behavior and extrusion stability [26]. Bioink concentration is another critical factor, and most studies report optimal printing performance and shape fidelity at concentrations between 2% and 4% [27]. Polymer blending is a widely used approach to further expand the functionality of alginate [26]. Composite bioinks containing alginate, time-aged oxidized cellulose nanofibers (TOCNFs), and polydopamine nanoparticles (PDANPs) combine the advantages of each component and show improved printability, mechanical strength, and osteogenic potential [28]. Similarly, the addition of carboxymethyl cellulose or montmorillonite clay has been shown to significantly enhance printing performance [29]. Among alginate modifiers, gelatin is the most commonly used thickener. Gelatin is frequently applied in cell-laden extrusion bioprinting due to its biocompatibility, bioactivity, and reversible gelation behavior. These properties have been extensively validated in both in vitro and in vivo studies [30].

Overall, alginate combines good biocompatibility, low cost, and easy gel formation under gentle conditions. These qualities make it one of the most versatile and widely used biomaterials in modern science and engineering [19].

#### 2.1.2. Hyaluronic Acid

**Figure 2:**
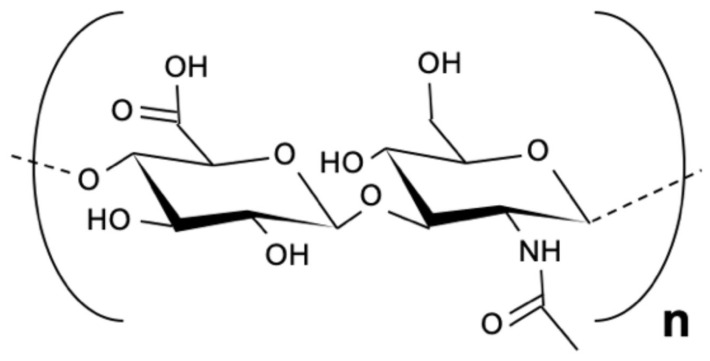
The structure of hyaluronic acid [36].

Hyaluronic acid (HA) is an anionic polysaccharide first isolated from the vitreous fluid of the eye by Karl Meyer in 1934 (Figure 2) [31]. Depending on its ionic form, it can also be called hyaluronate or hyaluronan. HA is a sulfate-free glycosaminoglycan that is naturally present in the synovial fluid, umbilical cord, and blood. Commercially, it is often produced through bacterial fermentation, especially using Streptococcus species [32]. Structurally, HA is a linear polymer made of repeating disaccharide units of β-1,4-linked D-glucuronic acid (GlcA) and β-1,3-linked N-acetyl-D-glucosamine (GlcNAc) [32]. Its molecular weight can vary widely, from 1000 to 10,000 kDa. High–molecular-weight HA is gradually broken down into smaller fragments, which can then enter the lymphatic system, bloodstream, liver, and kidneys [33]. HA contains several hydrophilic groups—hydroxyl, carboxyl, and acetylamino—that allow it to form both intra- and intermolecular hydrogen bonds and interact strongly with water molecules [34]. These interactions give HA excellent water solubility and hydration capacity. At the same time, the arrangement of hydrogen atoms creates small hydrophobic regions within the molecule, giving HA a flexible and dynamic structure in solution. Because of these properties, hyaluronic acid plays an important role in tissue hydration, wound healing, and angiogenesis. It also contributes to the structural framework of connective tissues. Its ability to interact with cells and extracellular components makes it a valuable biomaterial for applications in tissue engineering and regenerative medicine [35].

Natural hyaluronic acid shows excellent biocompatibility and strong bioadhesion, which makes it attractive for tissue engineering. However, HA degrades rapidly in vivo and has poor mechanical stability. These limitations restrict its direct use as a structural biomaterial. From a material perspective, HA has two key features. First, its native molecular structure is biologically passive [37]. Second, it contains functional groups that allow chemical modification. Because unmodified HA lacks shape retention, it is generally unsuitable for use as a bioink on its own. To address these issues, various modification strategies have been developed, including chemical crosslinking and grafting. In recent years, increasing attention has been given to physical blending approaches. For example, HA is often mixed with positively charged proteins or surfactants to improve network stability and structural integrity [38]. These strategies offer a simpler and more flexible way to enhance material performance without extensive chemical processing. The performance of HA-based bioinks in 3D bioprinting depends mainly on three interrelated factors: cell adhesion, mechanical integrity, and controlled degradation. These factors strongly influence cell survival, tissue growth, and long-term regeneration. Their combined regulation forms a core design principle in tissue engineering.

Cell adhesion plays a central role in regulating cell fate. Adhesion-mediated integrin signaling suppresses apoptosis and activates pathways such as PI3K–Akt, which promote cell survival and proliferation. Bioactive ligands, including RGD peptides, further guide stem cell differentiation. Mechanical integrity provides physical support and delivers mechanical cues through mechanotransduction pathways. Substrate stiffness directly affects cell behavior, while structural collapse or stress shielding can induce cell death. Degradation behavior must also be carefully controlled. Enzyme-sensitive matrices enable the timed release of growth factors and support angiogenesis, whereas uncontrolled hydrolytic degradation may trigger inflammation. Excessive degradation leads to loss of structure, while insufficient degradation limits cell infiltration and may cause fibrous encapsulation. The coordinated control of adhesion, mechanics, and degradation allows survival signals, lineage-specific cues, and tissue remodeling processes to act in concert. This synergy helps create a favorable microenvironment for regeneration. For optimal printability, both HA concentration and molecular weight must be precisely adjusted. Most studies report effective HA concentrations between 2% and 10% (*w*/*v*) and molecular weights ranging from 76 to 1550 kDa, as these parameters directly influence gelation rate and network density [39]. The optimal range varies with the printing method and target tissue. To further improve bioink performance, additional strategies have been explored. These include blending HA with polymers such as methoxy polyethylene glycol (mPEG), incorporating nanoparticles to tune flow and strength, and using gamma irradiation as a sterile and efficient crosslinking method. Together, these approaches enable the development of tailored HA-based bioinks that meet the mechanical, biological, and printing requirements of diverse tissue engineering applications while maintaining high bioreactivity.

#### 2.1.3. Xanthan Gum

**Figure 3:**
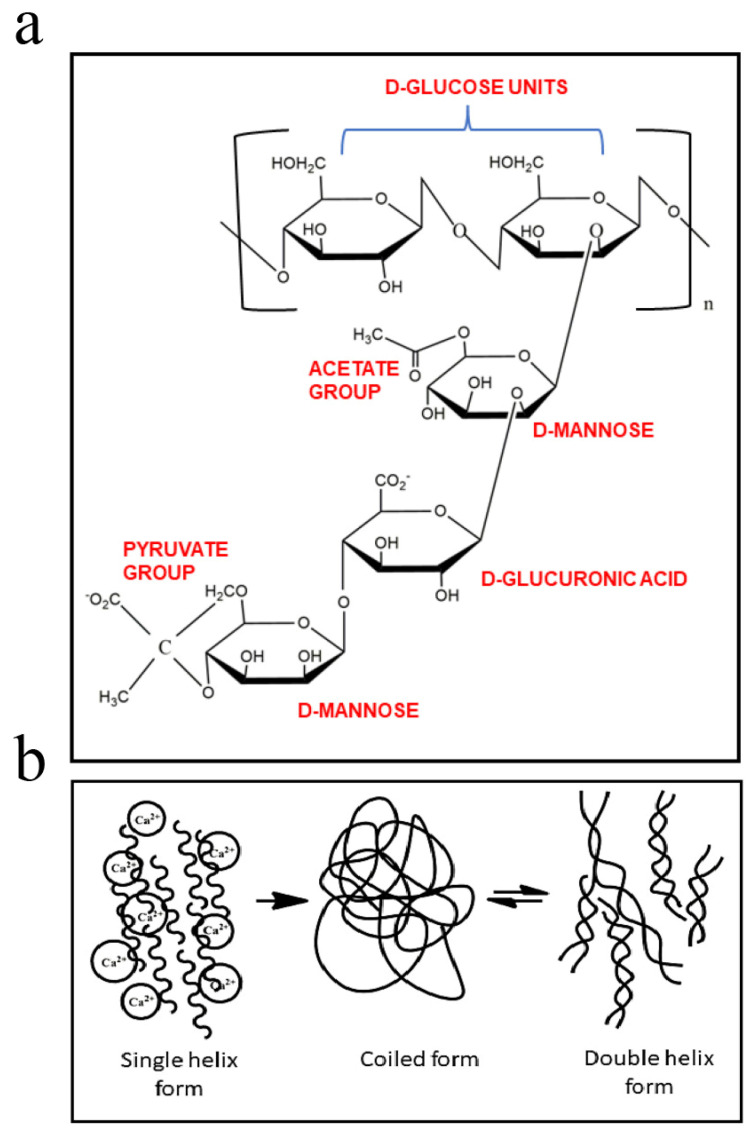
Molecular structure of XG−(**a**) and schematic illustration of the proposed mechanism of conformational ordering of XG−(**b**) [42].

Xanthan gum (XG) is an anionic exopolysaccharide produced by the fermentation of *Xanthomonas campestris* (strain NRRL B-1459). Its basic structure is built from pentasaccharide repeating units containing D-glucose, D-mannose, and D-glucuronic acid residues in a 2:2:1 ratio (Figure 3a,b). These sugar units may also include O-acetyl and pyruvyl side groups, which slightly modify the polymer’s properties. The main chain of xanthan gum consists of β-(1→4)-linked D-glucose residues, while the side chains attach to alternate glucose units [40]. The interaction between the main and side chains allows xanthan molecules to form a stable double-helix structure through hydrogen bonding.

This structure gives xanthan gum a strong and consistent viscosity even at low concentrations. The viscosity of xanthan solutions is sensitive to temperature, pH, and salt concentration. At low salt levels, electrostatic repulsion between the negatively charged chains causes the polymer to contract, reducing viscosity. When salt concentration increases, this repulsion decreases, and the molecular chains extend, resulting in higher viscosity [41]. Because of its stability and rheological properties, xanthan gum is widely used as a thickener, stabilizer, and emulsifier in the food, pharmaceutical, and cosmetic industries. In biomedical applications, it is also studied as a component of hydrogels and bioinks for tissue engineering, where its high viscosity and gel stability are valuable for supporting cell growth and controlled drug release.

Rheological properties, such as viscosity and shear-thinning behavior, play a central role in determining the bioprinting performance of bioinks. Experimental studies show that the static viscosity and shear-thinning behavior of xanthan gum solutions depend strongly on both polymer concentration and molecular weight [43]. When the concentration exceeds 2 g/L, viscosity increases exponentially with concentration [44]. The addition of complementary modifiers, including phosphate, sodium lactate, and ethyl lactate, reduces the dynamic viscosity of xanthan gum solutions, while the shear-thinning behavior remains largely unchanged. In salt-containing solutions, ionic screening neutralizes the negative charges on xanthan gum chains, which further alters gel rheology and flow behavior. Several studies have explored protein-assisted modification strategies to tune xanthan gum networks. Takuya reported that electrostatic attraction between xanthan gum and β-lactoglobulin (β-LG) can be induced during gel formation. In this system, β-LG aggregates along the xanthan gum chains and acts as a physical crosslinker [45]. By adjusting the β-LG–to–xanthan gum ratio, both the gelation kinetics and the final network structure can be precisely controlled, enabling tunable hydrogel formation. Xanthan gum also shows good compatibility with functional additives such as carbon nanotubes, graphene, and metal oxides [46]. Composite hydrogels containing these materials support the adhesion and proliferation of NIH 3T3 fibroblasts, particularly under magnetic stimulation. These findings highlight the potential of xanthan gum–based hydrogels for applications in skin, cartilage, muscle, and connective tissue engineering.

#### 2.1.4. Pectin

**Figure 4:**
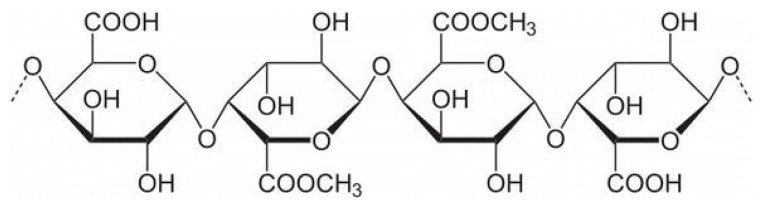
Pectin chemical structure [53].

Pectin is a natural anionic polysaccharide mainly found in the primary cell walls and middle lamella of higher plants [47]. It is abundant in fruits and vegetables and is commonly extracted from apple pomace, citrus peels, and sunflower heads [48]. Several methods are used to extract pectin, including acid extraction (using sulfuric, hydrochloric, or phosphoric acid), enzymatic extraction, supercritical carbon dioxide extraction, and ultrasound-assisted extraction [49]. The choice of method affects the yield, molecular weight, and gelling properties of the resulting pectin. Structurally, pectin is a complex polysaccharide composed mainly of α-1,4-linked D-galacturonic acid units (Figure 4). It includes three main structural regions: homogalacturonan (HG), rhamnogalacturonan I (RG-I), and rhamnogalacturonan II (RG-II) [50]. These regions differ in sugar composition and branching, giving pectin diverse physicochemical and biological properties. Pectin plays an important role in plant structure, providing rigidity and adhesion between cells. In the food and pharmaceutical industries, it is valued for its strong gelling, thickening, and stabilizing abilities [51]. In human health, pectin acts as a dietary fiber that supports gut function, reduces cholesterol absorption, and promotes intestinal peristalsis. It can also inhibit the adhesion of harmful microorganisms to the intestinal wall and has mild immunoregulatory effects [52].

Pectin with a low degree of methylation forms hydrogels in the presence of multivalent ions, such as calcium, magnesium, iron, and copper. In contrast, pectin with a high degree of methylation forms hydrogels mainly through hydrogen bonding and hydrophobic interactions under acidic conditions. Pectin hydrogels are non-toxic and have been used in several biomedical applications, including wound dressings, soft contact lenses, and drug delivery systems, as recently reviewed by Li et al. [54]. As a plant-derived polysaccharide, pectin has also gained attention as a component of bioinks for 3D bioprinting. Jovic et al. [55] highlighted its potential in reviews of plant-based biomaterials for 3D bioprinting, while Indurkar et al. [56] summarized its broader use in tissue engineering. These studies point to pectin as a promising and underexplored material in biofabrication. Hu et al. [57] investigated the incorporation of pure pectin into alginate–prullonate hydrogel bioinks to reduce inflammatory responses after cell replacement therapy. The resulting constructs were implanted in mice. The addition of pectin did not alter the viscoelastic behavior of the bioinks. The elastic modulus remained close to 190 kPa, which was similar to pectin-free formulations. Importantly, pectin improved the survival of bioprinted insulin-producing MIN6 cells under inflammatory stress. Cell viability increased to 73.3 ± 3.7%, compared with 64.4 ± 1.8% in constructs without pectin. These results highlight the ability of pectin to reduce inflammation while maintaining suitable mechanical properties. This feature makes pectin an attractive additive for future hydrogel bioink systems, especially in applications where immune response and cell protection are critical.

#### 2.1.5. Heparin and Heparan Sulfate

**Figure 5:**
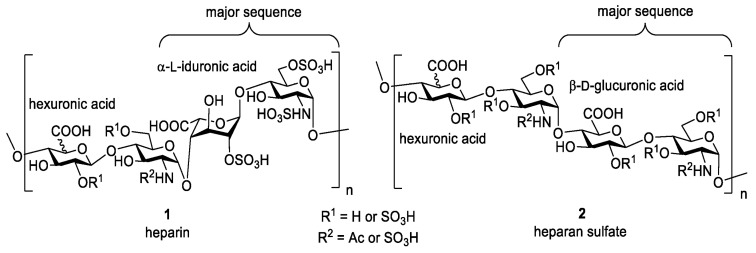
Structure of (**1**) heparin, (**2**) heparan sulfate [62].

Heparin is a naturally occurring glycosaminoglycan first discovered in the liver in 1916 [58]. It is composed of repeating disaccharide units of uronic acid and glucosamine linked by 1,4-glycosidic bonds. (Figure 5). Heparan sulfate (HS) is a structural analog of heparin, sharing a similar backbone but differing in sulfation patterns and tissue distribution [59]. Heparin is highly negatively charged due to its sulfate and carboxyl groups. This strong charge density leads to electrostatic repulsion between chains, giving the molecule an extended, non-coiled structure. It also allows heparin to bind selectively with many positively charged biomolecules, especially proteins and growth factors [60]. Chemically, heparin can be modified through esterification or amidation of its hydroxyl and amino groups to produce derivatives with specific biological or pharmacological properties [61]. One of heparin’s key features is its strong anticoagulant activity, which has made it one of the most widely used blood-thinning drugs in clinical practice.

Heparin is often selected for tissue engineering because it is low-cost, widely available, highly sulfated, and approved by both the FDA and EMA. Heparin is often used to improve the blood compatibility of biomaterials. When incorporated into scaffolds or hydrogels, it enhances hemocompatibility, reduces the risk of blood clot formation, and promotes the controlled release of heparin-binding growth factors such as VEGF, FGF, and TGF-β [63]. Heparan sulfate shares similar biological roles, participating in cell signaling, angiogenesis, and regulation of extracellular matrix interactions. Together, heparin and heparan sulfate are valuable in designing biomaterials that interact dynamically with cells and biological fluids. In bone tissue engineering, heparin-loaded delivery systems have been widely studied in both in vitro and in vivo models. These studies show that heparin can reduce the required dose of bone morphogenetic proteins (BMPs), such as BMP2, while improving their stability and biological activity. In many cases, heparin-containing biomaterials enhance BMP2 retention, controlled release, and osteogenic outcomes, leading to encouraging experimental results [64]. Despite these advantages, several concerns have emerged. At high concentrations, heparin can inhibit members of the transforming growth factor beta (TGF-β) superfamily, as well as other signaling proteins. In addition, the long-term side effects of heparin in tissue engineering applications remain unclear. Some heparin-based biomaterials have also failed to show positive effects in vivo [65]. A key limitation of heparin is its broad and non-specific binding behavior. While this property allows heparin to stabilize certain growth factors, it can also block other signaling molecules that are essential for tissue repair. This lack of selectivity may reduce treatment effectiveness or even lead to unwanted biological responses. For example, exogenous heparin has been shown to strongly inhibit growth differentiation factor 5 (GDF5), which plays an important role in cartilage and bone formation and tissue homeostasis. This finding is particularly concerning given the growing use of heparin in bone tissue engineering strategies. Overall, increasing evidence suggests that the use of heparin in clinical and tissue engineering applications should be approached with caution. These findings highlight the need to develop and use more specific glycosaminoglycans, such as heparan sulfate derivatives or synthetic analogs, which offer better structural control and fewer off-target effects for safer and more effective therapies.

#### 2.1.6. Polyglucuronic Acid (PGU)

Polyglucuronic acid (PGU) is a lesser-known but promising anionic polysaccharide composed of repeating D-glucuronic acid units. It can be obtained from various natural sources such as bacteria, algae, and fungi, each producing polymers with distinct structures and properties.

##### Bacterial PGU

**Figure 6:**
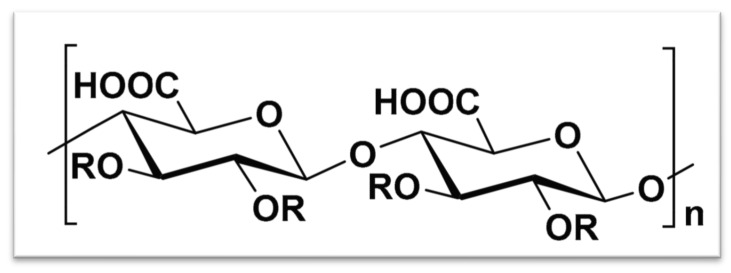
Structure of β-(1,4)-Polyglucuronic acid (PGU) from *Sinorhizobium meliloti* M5N1CS. With R=H or COCH_3_ (acetyl group) [22].

Certain bacteria, such as *Sinorhizobium meliloti* M5N1CS (NCIMB40472), produce polysaccharides composed primarily of glucuronic acid [66]. Gas chromatography analysis shows that this polymer is a homopolymer of β-(1,4)-linked D-glucuronic acid residues, often acetylated at the C2 and/or C3 positions (Figure 6). These bacterial PGUs display useful industrial properties such as gelling, thickening, stabilizing, and water-retaining abilities, making them valuable in food, pharmaceutical, and cosmetic applications. Further studies, including ^13^C NMR spectroscopy and molecular weight analysis, have confirmed the presence of unsaturated *β*-(1,4)-oligoglucuronic acid chains [67]. These oligosaccharides show good emulsifying behavior and maintain high thermal stability, which is beneficial for material processing and storage.

**Figure 7:**
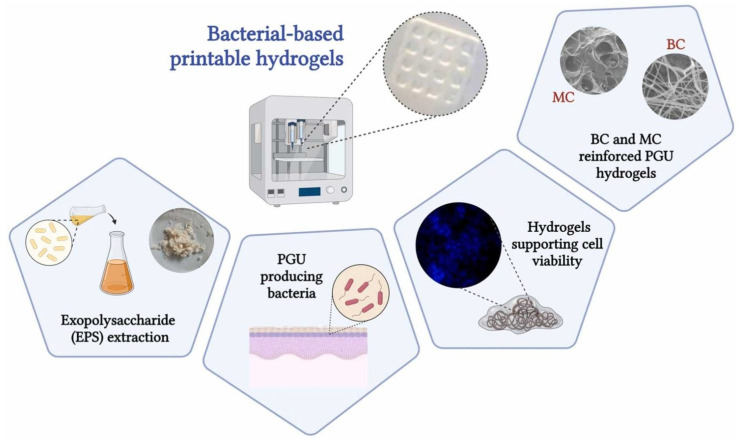
Development of printable biomaterial inks using a bacterial exopolysaccharide (EPS) called polyglucuronic acid (PGU), along with bacterial cellulose (BC) or methylcellulose (MC) [68].

Our laboratory has carried out several studies on bacterial polyglucuronic acid (PGU). We found that adding PGU to bacterial cellulose (BC) or methylcellulose (MC) inks improved the shape fidelity, printability, and stiffness of the 3D-printed hydrogels [68] (Figure 7). All cross-linked PGU-based hydrogels also showed good cytocompatibility with fibroblast cells (3T3-L1). These results suggest that PGU/BC or PGU/MC biomaterial inks have strong potential for use in tissue engineering, including injectable gels, drug delivery systems, and soft tissue models.

**Figure 8:**
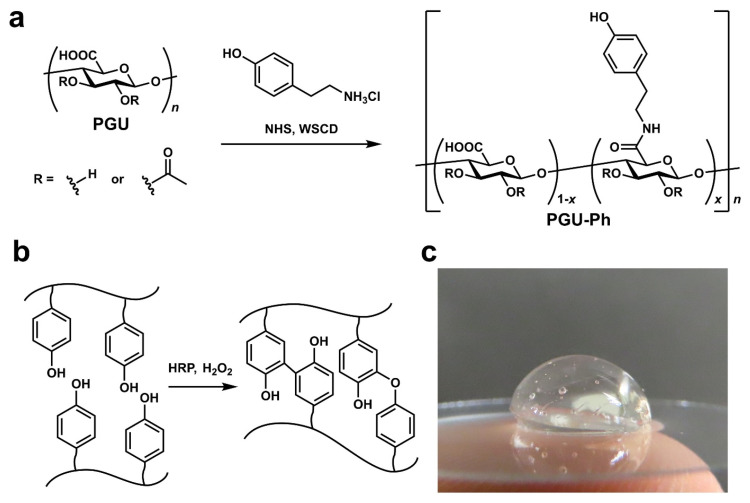
(**a**) Synthetic scheme of PGU-Ph, (**b**) cross-linking scheme of PGU-Ph through HRP-mediated reaction, and (**c**) photo of PGU-Ph hydrogel obtained through HRP-mediated reaction [69].

In addition, our study demonstrated that bacterial PGU could serve as an alternative to alginate for developing bioinks and other 3D printing materials. We further synthesized a phenol-grafted PGU derivative and examined its potential in tissue engineering as a bioink component for extrusion-based 3D bioprinting [69] (Figure 8). Aqueous solutions of this derivative were able to form hydrogels through a horseradish peroxidase (HRP)-catalyzed reaction. From a 2.0 *w*/*v*% solution containing 5 U/mL HRP, we successfully printed hydrogel structures that maintained high shape accuracy and stability.

Encapsulated mouse fibroblasts and human liver cancer cells showed about 95% viability one day after printing and remained viable for up to 11 days without significant loss of cell activity. These findings highlight the strong potential of PGU derivatives for tissue engineering, particularly as bioink components for extrusion-based 3D bioprinting. We also tested this phenol-grafted PGU using the sodium persulfate (SPS)/ruthenium photocrosslinking system, which produced gels with low cytotoxicity and promising mechanical properties.

##### Algal PGU

Polyglucuronic acid has also been identified in the cell walls of certain green seaweeds [70]. During extraction, algal glucuronic acid is often co-isolated with sulfated polysaccharides using hot water containing a calcium chelator such as sodium oxalate. This co-extraction can complicate the structural analysis of sulfated polysaccharides. To address this issue, researchers have employed ion-exchange chromatography to separate glucuronic acid components. Although effective, this technique is costly and time-consuming. More recently, a simpler method combining oxalic acid buffer extraction with acid precipitation has been developed. This approach yields high-purity glucuronic acid (around 94%) in just a few steps, as confirmed by NMR spectroscopy showing characteristic glucuronic acid signals [71].

##### Fungal PGU

In fungi, especially in mucormycetes, glucuronic acid–containing polymers are found in the cell wall matrix alongside chitin and chitosan. After extraction and chromatographic separation, calcium chloride can be used to precipitate these polysaccharides from solution, followed by acetic acid treatment to obtain purified fractions [72]. These polymers contribute to the mechanical strength and flexibility of the fungal cell wall. Overall, naturally derived PGUs—whether from bacteria, algae, or fungi—show potential as biopolymers for hydrogel formation, encapsulation, and other biomedical applications due to their high charge density, water solubility, and biocompatibility.

#### 2.1.7. Gellan Gum

**Figure 9:**
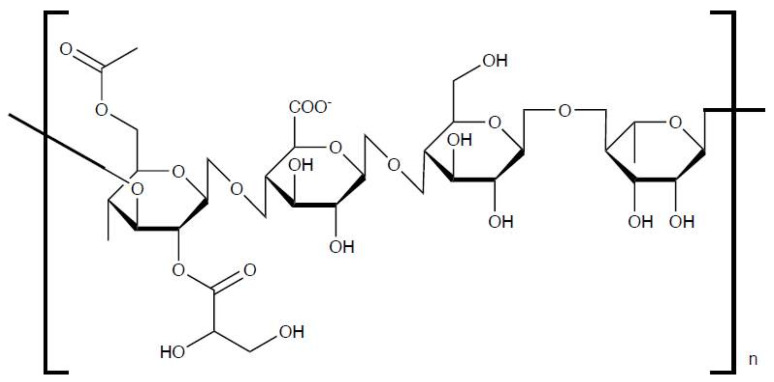
Chemical structure of gellan gum [73].

Gellan gum, a 500 kDa anionic extracellular bacterial polysaccharide, is produced by microbial fermentation of *phingomonas paucimobiis*. It consists of repeating units composed of β-(1,3)-D-glucose, β-(1,4)-D-glucuronic acid, β-(1,4)-D-glucose, and α-(1,4)-L-rhamnose [73]. This polysaccharide is also thermoresponsive, maintaining its coiled shape in solutions at temperatures above 60 °C. Below this temperature, it forms a hydrogel and transforms into a double-helix structure. Furthermore, due to the presence of carboxyl groups in the structure of gellan gum, it can form hydrogels in the presence of both monovalent and divalent ions (e.g., Na^+^, K^+^, Mg^2+^, and Ca^2+^). Because gellan gum hydrogels produced through ionic crosslinking are brittle and mechanically weak, they typically require chemical modification or combination with other polymers to form printable bio-inks [74]. Gellan gum (GG) (Figure 9), a widely studied microbial polysaccharide used in industries like food, pharmaceuticals, and cosmetics, plays a key role in creating bioinspired materials with promising health sector applications [75]. Although extensively researched, the clinical use of GG-derived materials remains in the early stages. Its impact on properties like thermo-mechanical characteristics, biocompatibility, and biodegradability has driven interest in its integration into various printing methods and formulations.

The shear-thinning behavior of gellan gum was first explored in 2014 for extrusion-based 3D bioprinting of living tissue constructs. In this approach, polylactic acid microcarriers loaded with mesenchymal stem cells (MSCs) were embedded in a gelatin methacrylate–gellan gum (GelMA–GG) hydrogel bioink. GelMA alone shows low viscosity and limited printability. The addition of gellan gum increased the viscosity of the solution and improved its printing performance [76]. Gellan gum helped the printed filaments retain their shape after extrusion. This effect enabled the fabrication of structures with high shape accuracy. Cell studies showed that more than 90% of MSCs remained alive three days after printing, and the cells were evenly distributed throughout the hydrogel. The printed scaffolds formed an open and porous network. This structure allowed efficient transport of oxygen and nutrients, which supported cell activity, as confirmed by integrated optical density (IOD) measurements. Similar strategies have been reported by other groups. For example, Zhuang et al. combined GelMA with agar to increase viscosity and improve both printability and shape fidelity [77]. However, GelMA–GG systems are often limited to printing simple and thin structures. To address this issue, Zhuang and co-workers developed a UV-assisted extrusion printing strategy. This method enabled the fabrication of more complex three-dimensional architectures. Through systematic printing tests, six GelMA–GG formulations were identified (5–0.5%, 7.5–0.1%, 7.5–0.2%, 7.5–0.5%, 10–0.1%, and 10–0.2% *w*/*v*). These formulations showed good print quality, stable filament formation, and effective cell encapsulation, with minimal cell settling. As expected, increasing polymer concentration led to higher mechanical strength. The compressive modulus increased from approximately 9 kPa to 16 kPa across these formulations. Beyond physical blending, gellan gum can also be chemically modified. Methacrylamide-modified gellan gum derivatives have been developed to enable photo-crosslinking and improved structural stability. Notably, a commercially available bioink based on GGMA is currently produced by AdBioInk, highlighting the growing practical relevance of gellan gum–based materials in 3D bioprinting.

#### 2.1.8. Carrageenan

**Figure 10:**
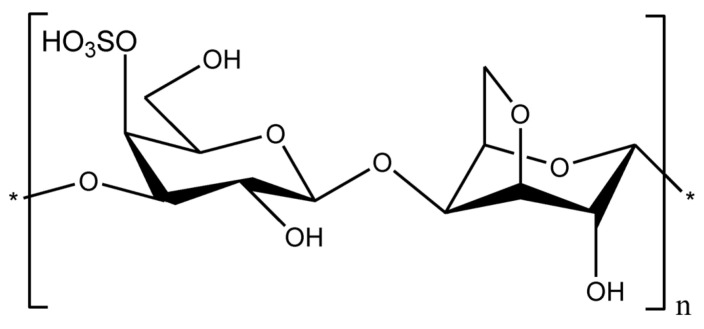
Chemical structure of κ-carrageenan [82].

Carrageenan is a natural anionic polysaccharide extracted from the cell wall of red seaweed [78]. Carrageenan is composed of β-1,3-D-galactose and α-1,4-D-galactose as the basic skeleton, alternately linked to form linear sulfate polysaccharides, which are divided into three main types according to the number of sulfate groups: Kappa (κ-), Iota (ι-) and Lambda (λ-) have one, two and three sulfate groups, respectively [79]. Carrageenan is generally a yellow or white powder, is odorless, and is a polymer compound; its molecular weight is not fixed. Commercial carrageenan molecular weights are usually between 100 and 1000 kDa. Different types of carrageenan exhibit different properties. Almost all types of carrageenan are soluble in hot water, and κ− is the strongest and most stable gel in water (Figure 10) [80]. Different types of carrageenan have a big difference in viscosity, generally carrageenan has a lower viscosity, while carrageenan has a higher viscosity due to more hydrophilic groups, but the viscosity of the three carrageenan is higher than that of agar gum. Due to the different sulfate content, the gel performance of the three carrageenan is completely different; Among them, ι-carrageenan and κ-carrageenan have good solidification, and when heated to boiling, they can melt to form sols, and continue to form gels after cooling, that is, a reversible sol–gel reaction, but λ-carrageenan generally cannot form a gel, that is, there is no gelling [79]. Compared to alginic acid, pectin and other colloids, carrageenan has good chemical stability. At pH below 4, heating carrageenan loses gel viscosity and strength due to breaking the ligation bond between 3,6 dehydrated-D-galactose. Carrageenan stability is reflected in the fact that it does not dissolve after a short period of heating under neutral conditions [81].

Compared to alginic acid, pectin and other colloids, carrageenan has good chemical stability. At pH below 4, heating carrageenan loses gel viscosity and strength due to breaking the ligation bond between 3,6 dehydrated-D-galactose. Carrageenan stability is reflected in the fact that it does not dissolve after a short period of heating under neutral conditions [81]. κ-carrageenan has been identified as an alternative for tissue engineering scaffolds due to its biocompatibility, low cytotoxicity, and excellent printability [83].

Carrageenan can form hydrogels in the presence of monovalent or divalent cations, such as potassium or calcium ions. This gelation occurs through interactions between the cations and the sulfate groups on the carrageenan chains. As with many hydrogel systems, both the gelation behavior and final viscosity depend on several factors. These factors include the type of carrageenan, sulfate content, molecular weight, polymer concentration, and temperature [84]. Carrageenan-based hydrogels are well established in biomedical applications. Previous studies have reported their use in drug delivery, tissue engineering, and wound healing [85]. More recently, researchers have also highlighted their potential as components of bio-inks for 3D bioprinting. However, native carrageenan hydrogels often show limited printability and weak mechanical stability under physiological conditions [86]. To overcome these limitations, various modification strategies have been explored. One common approach is the incorporation of nanostructures, such as nanosilicates (nSi), to reinforce the hydrogel network [87]. Another strategy involves blending carrageenan with other biopolymers, including gelatin and alginate [88], to improve rheological behavior and structural integrity. Chemical modification of the carrageenan backbone has also been widely investigated, particularly the introduction of methacrylamide or methacrylate groups to enable photo-crosslinking [89]. Mihaila et al. first reported the chemical modification of κ-carrageenan with methacrylate groups to create hydrogels using a dual crosslinking strategy [90]. In this system, the bio-ink was first exposed to ultraviolet light and then treated with potassium ions. The resulting hydrogels showed good extrusion printability. Scaffolds loaded with human mesenchymal stem cells (hMSCs) maintained structural stability and supported cell viability for up to 21 days, with approximately 75% viable cells. In a later study, methacrylamide-modified κ-carrageenan (1% *w*/*v*) was combined with GelMA (10% *w*/*v*) to fabricate 3D-printed scaffolds for adipose tissue regeneration, with a focus on breast reconstruction applications [89]. The bio-ink, containing adipose-derived stem cells (ASCs), was printed by extrusion and crosslinked using UV light. The resulting scaffolds exhibited mechanical properties close to those of native breast tissue, with a Young’s modulus of approximately 2 kPa. The constructs remained stable for up to 21 days and supported high cell viability (>94%) as well as enhanced cell proliferation (>128%) over 14 days. At present, methacrylamide-modified carrageenan represents the most mature formulation for bioprinting applications. To our knowledge, the only commercially available carrageenan-based bio-ink, KapMA, is derived from methacrylamide-modified carrageenan and is produced by AdBioInk.

We compared the advantages and disadvantages of the above eight anionic polysaccharides (Table 1). Based on existing literature, we propose potential future applications of these materials in tissue engineering. According to tissue engineering needs, we recommend the following: (1) High shape fidelity & printability: Xanthan gum, gellan gum, PGU; (2) High bioactivity & cell interaction: Hyaluronic acid, heparin (low dose), modified alginate; (3) Inflammation control: Pectin, HA; (4) Fast and mild gelation: Alginate, carrageenan, PGU; Growth factor delivery: Heparin, HA-based hybrids. Most high-performance bio-inks rely on hybrid systems, where one polysaccharide provides mechanical integrity or rheological control, while another contributes biological signaling or degradation control. Chemical modification (oxidation, methacrylation, phenol grafting) and multi-step crosslinking (ionic + enzymatic or photo) are increasingly used to tailor inks to specific tissue engineering needs.

### 2.2. Preparation of Anionic Polysaccharides by Modification

Synthetic anionic polysaccharides are engineered materials designed to overcome the limitations of their natural counterparts, such as uncontrolled degradation, poor solubility, and inconsistent molecular weight. Consequently, modifying natural polysaccharides (cellulose, for example) to acquire the properties of anionic polysaccharides can mimic the synthesis process of these materials. Broadly, polysaccharide surface modification methods can be categorized into three approaches: (1) Surface Chemistry: Modifications derived from polysaccharide extraction or treatments applied to the polysaccharide surface using similar techniques. (2) Adsorption: The physical adsorption of molecules onto the cellulose surface. (3) Covalent Binding: The covalent attachment of molecules or derivatization of the natural polysaccharide surface. These modification strategies are typically adapted from the pulp and paper industry, utilizing the inherent functional groups of polysaccharides, such as hydroxyl groups, as “handles” for further modification. Since cellulose is the most widely distributed natural polysaccharide and the current research focus is on the modification based on cellulose, the following synthesis of anionic polysaccharides is mainly carried out using cellulose as an example.

#### 2.2.1. Functionalization by Adsorption

**Figure 11:**
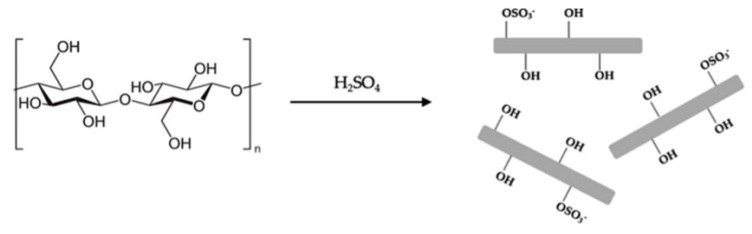
Cellulose hydrolysis by sulfuric acid [106].

The first modification method involves adsorption onto the polysaccharide surface. Typically, electrostatic interactions are employed to stabilize nanoparticles with surfactants. Since nanoparticles tend to exhibit poor dispersion in organic media and polymers, surfactants are commonly used for stabilization. Polysaccharide obtained through sulfuric acid hydrolysis possesses a charged surface (Figure 11), which facilitates the adsorption of commonly used surfactants, such as stearic acid, cetyl bromide, and trimethylammonium bromide [102]. Another widely used approach for adsorption-based modification is the electrostatic adsorption of macromolecules, a method adapted from the papermaking industry. Since cellulose is known to carry a very weak charge, polyelectrolytes have been employed as dry and wet strength additives, antistatic agents, and for other purposes. The layer-by-layer deposition method is a frequently utilized technique for this purpose. Additionally, nonionic adsorbents and dispersants have also been applied in cellulose modification [103,104]. Xylan exhibits strong obligate adsorption onto cellulose surfaces, and xylan block copolymers function as effective nonionic adsorbents and dispersants [105]. Cellulose nanofibers prepared via TEMPO oxidation possess a highly charged surface, making them well-suited for adsorption-based modification methods.

#### 2.2.2. Functionalization by Cellulose

Cellulose does not dissolve in water. This property limits its direct use in many biomedical applications. Researchers often modify the cellulose structure to create water-soluble derivatives with strong affinity for water. Scientists usually introduce hydrophilic groups, such as carboxyl groups, into the polymer chains to achieve this goal. Researchers can classify cellulose derivatives into three main types based on their chemical structure. These types include cellulose ethers, cellulose esters, and cellulose ether esters. Common cellulose derivatives include carboxymethyl cellulose (CMC), hydroxypropyl methyl cellulose (HPMC), hydroxyethyl cellulose (HEC), and hydroxypropyl cellulose. Among these materials, methyl cellulose (MC) has been widely used to prepare hydrogels for multilayer bioprinting [107]. The synthesis of carboxymethyl cellulose from cellulose follows a classic chemical pathway. The reaction is based on the Williamson ether synthesis principle. Many industrial methods exist for producing CMC. The most common method is the solvent process, which usually occurs in an organic solvent system. The slurry method with a high liquid ratio is especially popular. This method provides efficient mass transfer and heat transfer. This method also produces a uniform distribution of substituent groups along the cellulose chains. Because of these advantages, industries widely use this approach to manufacture medium- and high-grade CMC [108]. Sulfonate groups can form on the cellulose surface through sulfuric acid degradation, imparting a high acid content to the surface. Intense treatments, such as prolonged reaction times, result in a high degree of sulfonation. In contrast, hydrochloric acid degradation is less commonly employed and leads to the formation of hydroxylated surfaces [109]. Other, less commonly used methods, such as hydrolysis of phosphoric acid and hydrobromic acid [110]. It was also reported. Using acetic acid as an acid catalyst, Fischer esterification can be carried out to acetylate the cellulose surface [111]. Sulfuric acid degradation is the most commonly used method, owing to its ability to produce cellulose with a high sulfuric acid content, high surface charge density, and enhanced stability of the nanocrystalline suspension. Consequently, some researchers employ post-sulfonation to further increase the density of sulfonic acid groups on the surface of the product [112]. Various methodologies can be employed to extract nanocellulose, specifically microfibrillated cellulose (MFC) and nanofibrillated cellulose (NFC), each exhibiting distinct fiber properties and surface chemistries. The purely mechanical processes, which encompass steam explosion, high-pressure homogenization, and high-shear mixing, do not involve oxidation or degradation mechanisms, thereby yielding cellulose that closely resembles natural cellulose, characterized by a hydroxylated surface. Alternatively, the utilization of 2,2,6,6-tetramethylpiperidin-oxide (TEMPO) for controlled oxidation, in conjunction with low-shear mechanical treatment, is increasingly gaining traction in the field [113]. This method involves the use of TEMPO (2,2,6,6-tetramethylpiperidin-1-oxyl) radicals as catalysts, along with primary oxidants such as hypochlorite, to selectively oxidize the hydroxyl groups located on the C_6_ position of the cellulose molecular chain. The oxidation process enhances the breakdown of cellulose feedstocks, making it easier to mechanically process the material at low speeds, thereby facilitating the dissociation of fiber bundles into nanofibers. The selective oxidation of the hydroxyl groups results in the formation of carboxyl groups on the surface of the nanocellulose. This modification not only alters the chemical properties of the nanocellulose but also improves its compatibility with various matrices, potentially enhancing its application in composite materials, coatings, and other advanced materials. The presence of carboxyl groups can also provide additional sites for further functionalization, reinforcing the versatility of nanocellulose in various industrial applications.

#### 2.2.3. Functionalization by Chemical Modification

The third method of surface chemical modification involves direct chemical modification or covalent bonding of molecules. Given that cellulose possesses numerous hydroxyl groups on its surface, it can react with alcohols such as isocyanates, epoxides, acid halides, and anhydrides. These reactions yield a variety of surface groups, including amines, ammonium, hydroxyls, esters, and acids. Utilizing nanofiber preparation technology, the hydroxyl groups can be transformed into a base using nanoparticles hydrolyzed by TEMPO oxidative acid to achieve improved dispersion [114]. Other techniques for modifying the surface of cellulose, such as sulfuric acid treatment, can also produce sulfonates. For cellulose nanocrystals, these methods generally enhance their dispersion in organic solvents and polymer resins. This improved dispersion can further enhance the mechanical properties of the resulting composites. Beyond the straightforward application of surfactants or alkylation reactions, many researchers employ covalent bonding to attach polymers to the surfaces of nanoparticles, thereby improving compatibility with resins like malayan polyolefins. For instance, polypropylene can be bonded to the surface of the nanocrystals through anhydride modification [115]. In addition, the method of grafting polymer monomers on the surface of cellulose and then polymerizing can also be employed [116]. Recently, biodegradable cellulose nanocrystalline/polycaprolactone biocomposites have been prepared by using surface-grafted polycaprolactone as a solubilizer and a surface hydroxyl group and a chemical agent as initiators [117]. Utilizing the hydroxyl group on nanocellulose as a nucleophile facilitates its carboxymethylation. Furthermore, polymers can be grafted onto the surfaces of dissociated fibers to enhance their compatibility in composites, while alkoxysilanes and chlorinated silanes can also be employed to modify the surface of cellulose through silanization [118]. Silanes are covalently bonded to cellulose.

**Figure 12:**
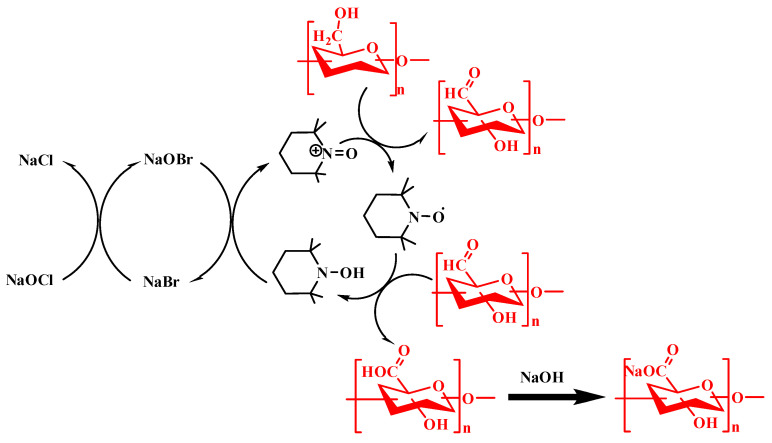
Mechanism of TEMPO-mediated oxidation of cellulose [123].

TEMPO oxidation enables the selective conversion of primary hydroxyl groups in polysaccharides into carboxyl groups. This introduces controlled charge and improves water solubility—key parameters for bio-ink formulation. The resulting polymers show enhanced dispersion, tunable viscosity, and improved printability [119,120] (Figure 12). TEMPO-modified polysaccharides form ionic and physical gels. Carboxyl groups enable crosslinking (e.g., with calcium ions), supporting rapid gelation and structural stability after extrusion. This is critical for shape fidelity in 3D bioprinting. The modification also promotes shear-thinning behavior, which facilitates smooth extrusion and recovery [121]. Oxidation disrupts crystalline regions and reduces molecular weight. This improves polymer chain mobility and rheological control. Increased water uptake further enhances hydration and cell-compatible environments in bio-inks [122,123]. TEMPO-treated cellulose and starch can form stable, biocompatible hydrogels. These materials support cell encapsulation, scaffold formation, and controlled release. Functional groups introduced during oxidation also allow further biofunctionalization [124,125,126,127]. Different TEMPO systems enable control over oxidation level and polymer integrity. Neutral and enzymatic systems better preserve chain structure, which is important for mechanical performance in printed constructs. Electrochemical methods offer cleaner processing routes with fewer residues, improving biocompatibility [128,129]. TEMPO modification provides a precise and scalable way to tune charge density, solubility, rheology, and crosslinking behavior of polysaccharides. This makes it a central strategy for producing printable, stable, and cell-compatible bio-inks. We also noted several other chemical modification methods, such as methacrylation, sulfonation, and thiolation. These approaches are widely used to adjust the properties of polysaccharides so they can achieve better printing behavior and improved mechanical strength. Such modifications help overcome the natural limits of polysaccharides when they are used in soft tissue regeneration. By effectively modifying and crosslinking these polymers, researchers can also fine-tune their biological functions. This allows better control over cell behavior and supports key processes involved in tissue repair. As a result, these modified polysaccharides can help promote the regeneration of soft tissues such as skin, cartilage, muscle, and nerve tissue [7].

#### 2.2.4. Functionalization by Amino Acid

Biomaterials can form networks through ionic, chemical, or physical cross-linking. Researchers can also modify these materials with amino acids to improve biocompatibility and stability. This strategy helps overcome the natural limitations of some polymers. Cysteine is a naturally occurring amino acid that contains a thiol group on its side chain. This thiol group has the structure −SH and contains one sulfur atom and one hydrogen atom. The sulfur atom in the thiol group is nucleophilic. This property allows it to react with electrophilic groups such as carbonyl groups and alkyl halides. The reactivity of cysteine increases when the thiol group becomes ionized. When the thiol loses a proton, it forms a thiolate anion. The sulfur atom in the thiolate form is more reactive in nucleophilic reactions. This increased reactivity enhances its participation in cross-linking reactions and catalytic processes. Cysteine cross-linking provides a feasible and relatively safe chemical method for building soft tissue engineering scaffolds. Peptides that contain high levels of cysteine can undergo several types of reactions. These reactions help improve scaffold performance. One important advantage is that cysteine-based reactions are not limited to a specific polymer type. Researchers can apply this chemistry to many different polymer systems. Cysteine often forms disulfide bonds during cross-linking. These disulfide bonds provide mechanical strength and structural stability. These bonds are also reversible under certain conditions. This reversibility supports controlled degradation and improves biodegradability. Cysteine contains three main functional groups: amino (-NH_2_), carboxyl (-COOH), and thiol (-SH). These groups give cysteine favorable physicochemical and biological properties. These properties support good biocompatibility and make cysteine suitable for soft tissue scaffold design. Cysteine can also regulate growth factor release through disulfide bond exchange reactions. Researchers can use these reactions to modify scaffold surfaces and promote cell signaling. This modification increases the biological activity of the scaffold. In addition, thiol groups can enhance polymer functionality by forming reactive thiolate species that participate in further cross-linking or surface modification reactions [130].

## 3. Advantages of Anionic Polysaccharides as Hydrogel Bioinks

Hydrogels are getting more attention because they can be designed with different chemical, mechanical, and biological features. They also allow cells to be safely held inside the material. These networks can be made in several ways, including enzyme-based reactions, hydrophobic interactions, ionic bonding, and changes in temperature or light. Anionic polysaccharides are among the most commonly used materials for constructing bioinks in bioprinting, owing to their excellent printing performance, rapid cross-linking capability with calcium ions, and good biocompatibility, which facilitates cell encapsulation. Their superior printing performance is primarily attributed to their ability to form hydrogels through cross-linking with calcium ions, enabling the bioink to maintain its mechanical structure during and after printing. Crosslinking is carried out by physical or chemical methods to make a hydrogel scaffold that meets the requirements

### 3.1. Physical Crosslinking

**Figure 13:**
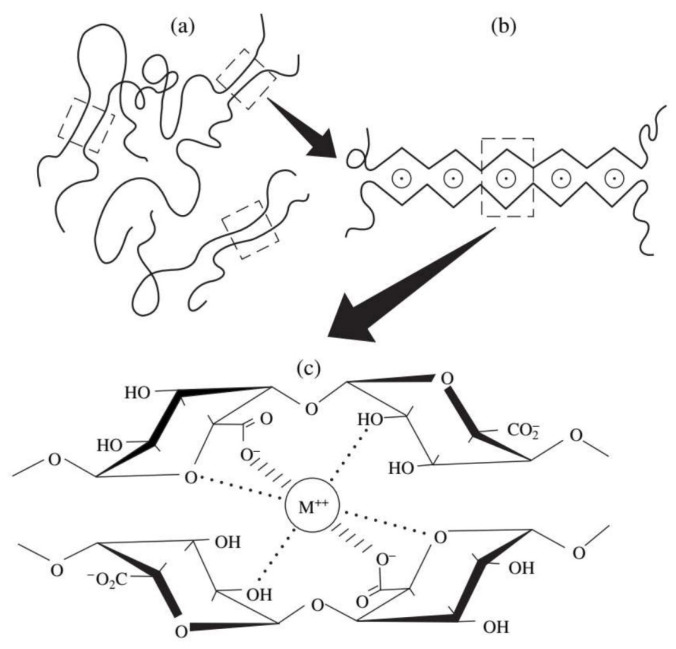
The conformation of monomers and block distribution of alginate. (**a**) Macrostructure of alginate chains, (**b**) alginate egg-box gelation model with divalent cations, (**c**) Chemical structure diagram after b alginate is cross-linked with ion. Adapted from [140].

Physical crosslinking is typically accomplished through intermolecular forces, including ionic bonds, crystal recombination forces, hydrophobic or hydrophilic interactions, and metal coordination bonds. Since no chemical crosslinking agents are introduced, the primary advantage of physical crosslinking is its non-toxic nature [131]. Alginic acid, hyaluronic acid and pectin are all active anionic polysaccharides, and special functional groups in their molecular structure such as carboxyl groups, amines and sulfates can be crosslinked with various divalent ions or trivalent ions (such as Ca^2+^, Sr^2+^, Zn^2+^, Fe^3+^) (Figure 13) [132]. For example, people use anionic polysaccharides to crosslink with counterions of tripolyphosphate (TPP), which is used in various biomedical projects [133]. Thin films, hydrogels, microspheres, and other materials composed of physically crosslinked alginate and cationic polysaccharide oligomers have demonstrated utility in drug delivery and tissue regeneration [134]. Alginate can react with a variety of divalent metal ions. Because calcium ions are not cytotoxic, they are often used in the preparation of calcium alginate. The hydrogel resulting from the interaction between alginic acid and calcium ions will exhibit enhanced structural stability [135]. Polysaccharide-based hydrogels can also undergo self-assembly through the electrostatic interaction of ionic polysaccharides bearing opposite charges. This self-assembly is achieved by ionic strength and pH control [136]. For instance, dextran self-assembly and miRNA-loaded hydrogels have found applications in drug delivery [137]. Moreover, successful self-assembly of hyaluronic acid and its derivatives into β-cyclodextrin and adamantane hydrogels has been achieved [138]. Furthermore, recent studies have delved into the condensation and complexation mechanisms of polysaccharides, with the complexation process being driven by entropy. For instance, hydrogels are self-assembled through interfacial polyion complexation involving chitosan, alginate, and carrageenan. These hydrogels can have their tensile strength adjusted by incorporating cerium oxide nanoparticles [139]

Chitosan forms cross-linked networks when combined with polyanions like tripolyphosphate (TPP) through ionic gelation. This occurs when positively charged amino groups on chitosan molecules interact with negatively charged TPP ions. The resulting ionic bonds create a physically cross-linked hydrogel structure without requiring chemical cross-linking agents [141]. A research team developed an innovative 3D printed scaffold combining chitosan, gelatin, tricalcium phosphate, and graphene oxide. After printing, they treated the scaffolds with l-ascorbic acid, which converted the graphene oxide into its reduced form within the structure. The researchers then crosslinked these scaffolds by soaking them in a tripolyphosphate (TPP) solution. The reduced graphene oxide-containing scaffolds showed particularly promising results, suggesting potential advantages for bone tissue engineering applications. The TPP crosslinking method effectively enhanced both the physical and biological properties of the composite material while preserving its structural integrity [142]. Polyelectrolyte complexes form through electrostatic attraction between positively and negatively charged molecules. In acidic environments, chitosan acts as a positively charged polymer with strong charge properties. It readily binds with negatively charged polymers like alginate, gelatin, dextran sulfate, and pectin. The complex formation works best when some of the charges on both chitosan and the polyanions are neutralized. This partial neutralization allows the molecules to come together and form stable complexes without fully losing their charged properties. The balance between attraction and repulsion forces helps create these useful structures for various biomedical applications. This process provides a simple way to combine different polymers while maintaining their beneficial characteristics. The resulting complexes often show improved properties compared to the individual components alone [141].

### 3.2. Enzymatic Crosslinking

Enzymatic oxidation has become a powerful and eco-friendly strategy for the functionalization and crosslinking of polysaccharides. Among the commonly used oxidases, tyrosinase, laccase, and peroxidase catalyze oxidative coupling reactions that generate reactive radicals or quinones, enabling polymerization or hydrogel formation under mild and biocompatible conditions.

**Figure 14:**
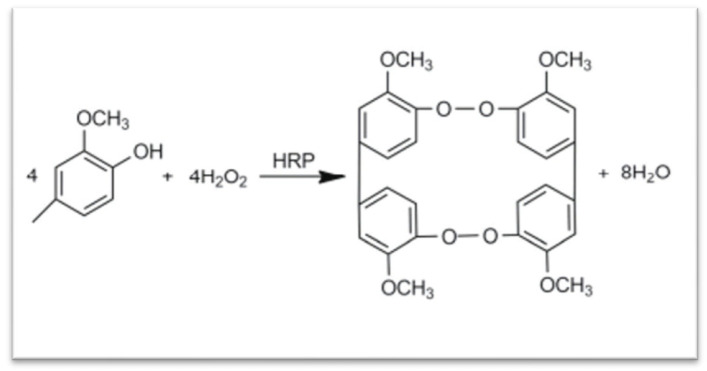
Oxidation of guaiacol by H_2_O_2_ catalysed by HRP.

Tyrosinase, a copper-containing enzyme, catalyzes the ortho-hydroxylation of monophenols to o-diphenols and their subsequent oxidation to o-quinones. These quinones readily react with nucleophilic groups on polysaccharides to form covalent crosslinks. Tyrosinase-based systems are simple and oxygen-driven but limited to phenolic substrates and are sensitive to pH and inhibitors. Laccase is a multicopper oxidase that performs one-electron oxidation of phenols or anilines using molecular oxygen as the electron acceptor, producing water as the only byproduct. The generated phenoxy radicals undergo spontaneous coupling to form C-C or C-O linkages. Laccase offers high substrate versatility and environmental compatibility, though its reaction rate is often constrained by oxygen solubility and diffusion limitations. Horseradish peroxidase (HRP) is a key enzyme used for mild crosslinking reactions in polymer systems [143]. When added to a polymer–phenol solution with hydrogen peroxide, HRP catalyzes covalent bond formation, producing a stable hydrogel network. The gelation process occurs under gentle conditions—neutral pH and room temperature—making it suitable for cell encapsulation and tissue engineering. The gelation time depends mainly on HRP concentration, while H_2_O_2_ controls the mechanical strength of the resulting hydrogel. HRP-crosslinked gelatin or alginate hydrogels rich in collagen can support cell adhesion, differentiation, and extracellular matrix deposition, providing ideal environments for chondrogenic and osteogenic tissue formation (Figure 14). Subsequent radical coupling between phenol-modified polysaccharide chains yields covalent crosslinks and hydrogel networks. The HRP/H_2_O_2_ system allows rapid, tunable polymerization under physiological conditions, making it highly suitable for injectable hydrogels, tissue engineering, and drug delivery. However, excessive H_2_O_2_ can inactivate the enzyme or cause oxidative degradation. Overall, peroxidase-mediated polymerization offers superior control over reaction kinetics and gelation behavior, positioning it as the most versatile oxidase system for designing enzyme-crosslinked polysaccharide hydrogels in biomedical applications.

Hydrogels formed through chemical crosslinking exhibit enhanced stability and a broad spectrum of mechanical properties, including increased tensile strength, shear stress resistance, and bending capabilities. Current chemical crosslinking methods encompass enzymatic-induced crosslinking, oxime formation, free radical polymerization, Schiff base formation, Diels–Alder “click” reactions, and Michael reactions [144]. The Michael addition reaction involves the interaction between two components, one possessing an electrophilic group and the other a nucleophilic group, resulting in the formation of a chemical crosslink [145]. Click chemistry is also extensively employed in designing crosslinked hydrogels due to its versatility in accommodating various functional groups, the mild reaction conditions it offers, its high chemical selectivity, and its ability to yield high-quality products [146]. To circumvent the introduction of toxic metals, researchers have explored numerous metal substitutes. One such approach involves employing dialoxylic acid chemistry to combine furan-modified alginate with bismaleimide crosslinkers [147]. Furthermore, the formation of Schiff bases offers another avenue for creating biocompatible hydrogels for tissue engineering. These hydrogels can be readily prepared by reacting hydroxyl (OH), amino, or hydrazide functional groups with aldehyde groups under mild reaction conditions [148]. For example, Schiff bases can be formed through reactions involving polyaldehyde glucan and carboxymethyl chitosan [149]. Vinyl radical polymerization represents another chemical modification approach for anionic polysaccharides. In this process, the initiator decomposes into free radicals, which subsequently react with double bonds to facilitate polymerization. Controlling the initiator concentration and temperature plays a crucial role in achieving the desired target product [150]. In contrast to physical crosslinking, chemical crosslinking offers a notable enhancement in the mechanical properties and stability of hydrogels. The broad selectivity of chemical crosslinking agents expands the range of potential applications for these hydrogels. However, it’s important to note that, besides potential toxicity concerns, chemical crosslinking can introduce color to biological materials, which may restrict their suitability for use in tissue engineering.

### 3.3. Photo Crosslinking

Common chemical cross-linkers for cationic polysaccharides include genipin, glutaraldehyde, and EDC/NHS. Glutaraldehyde is frequently used to cross-link chitosan, but it can be toxic to cells. On the other hand, genipin is much less toxic and offers additional benefits like antibacterial and anti-inflammatory effects. This makes genipin a safer alternative for biomedical applications where biocompatibility matters. The choice between these cross-linkers often depends on balancing strength requirements with safety considerations [141,151]. Chitosan-based printed film matrices were created for wound dressings. Researchers cross-linked these matrices with genipin and used polyethylene glycol (PEG) as a plasticizer. This process led to a slower release of drugs and a delay in degradation. The formation of strong, irreversible bonds improved the overall stability and strength of the dressings [151]. Another study added NHS/EDC to a mixture of collagen and chitosan hydrogels before printing. This combination resulted in stronger gels that had better mechanical properties and higher viscosity [152]. Photopolymerization improves the mechanical strength of hydrogels. This process helps prevent the gels from collapsing during printing [141]. The researchers prepared chitosan-based bioinks using a combination of light and heat to form stable gels. First, thermal crosslinking helped maintain the printed structure’s shape. Then, UV light at 365 nm strengthened the material further through photocrosslinking. They combined methacrylated chitosan with β-Phosphoglycerate to improve temperature sensitivity, allowing for high-precision printing. The resulting bioink maintained structural integrity without damaging cells during the printing process, demonstrating good biocompatibility [153].

#### 3.3.1. The Principle of Photo Crosslinking

Using light-based methods to form hydrogels works especially well for additive manufacturing and 3D printing. These methods offer built-in control over where and when the crosslinking happens, which helps improve the printing process and build precise 3D structures. The polymerization of cross-linked materials through radical chains usually happens in three steps: initiation, propagation, and termination. Each step has its own reaction speed, which can affect both the small-scale structure and the overall network of the material. In radical chain polymerization, the process starts by creating free radical species. During the initiation phase, a photoinitiator is broken down by light—through photolysis or photo-induced cleavage—into reactive radicals. The speed at which these radicals form depends on several factors: (I). The intensity of the light used. (II). How effective the photoinitiator is. (III). The amount of photoinitiator present. (IV). The quantum yield of the light reaction. (V). The number of useful radicals made per photolysis event (for example, homolysis usually creates two active radicals) [154]. After the initiation step, the free radicals react with certain functional groups on the polymer chains. This forms new covalent bonds and creates active radical intermediates. These intermediates can then continue to react with other available groups, allowing the radical species to spread and form growing polymer chains. Propagation mainly involves free radicals reacting with unreacted double bonds, such as those in methacrylates, acrylates, or acrylamides. Since most of these reactions happen during the propagation stage, the loss of double bonds can be treated as a secondary reaction in the overall process. The propagation process eventually stops during the termination phase of the reaction. Termination can happen in a few different ways. One common way is radical coupling, where two growing chain ends join to form a single, longer chain. Another is disproportionation, where two chain ends stop growing—one ends with a saturated group, and the other with an unsaturated group. Termination can also occur through chain transfer, where the free radical is passed to another molecule, pulling it away from the growing polymer chain and stopping the reaction. The kinetics of radical chain polymerization have been widely studied using pulsed laser polymerization combined with size exclusion chromatography (PLP-SEC) [155], especially for common functional groups like acrylates and methacrylates. Research shows that acrylates usually polymerize faster than methacrylates [156]. The reactivity of these groups can also be tuned. In general, acrylates and methacrylates that have electron-withdrawing groups nearby react more quickly. These groups help stabilize the intermediate radical species during polymerization. Also, acrylates and methacrylates with longer alkyl chains tend to be more reactive, making chain length another factor that influences the speed of the reaction. Tracking the mechanical properties of hydrogels is a common way to study how the reaction progresses and how liquid precursors like bioinks or bioresins turn into solid gels. One popular method is oscillatory shear rheology, which measures changes in material properties in real time. In this method, the storage modulus (G′) shows how elastic the material is, while the loss modulus (G″) reflects its viscous behavior. For example, when methacrylated hyaluronic acid (MeHA) is cured using UV light and the photoinitiator Irgacure 2959, the polymerization rate increases with stronger light and higher photoinitiator concentration. This shows how key parameters like light intensity and initiator level can control the reaction speed in bioinks and bioresins. Fine-tuning these factors is essential to match the needs of specific bioprinting methods or even individual bioprinters. The structure and crosslinking density of the light-cured network formed by radical chain polymerization can be adjusted by changing the initiator concentration, the amount of active functional groups, and the light intensity. In general, a higher crosslinking density leads to stronger mechanical properties and slower degradation, especially for materials designed to break down over time [157]. The chain length distribution created during propagation also adds variability to the local network structure. This happens because rapid chain formation can create concentration gradients and diffusion limits, which speed up propagation but slow down radical termination. During polymerization, as the network forms, steric hindrance makes it harder for radicals to meet and terminate. This raises the total number of free radicals and causes an automatic speed-up in the reaction. Near the end, the reaction slows down again because radicals cannot move as freely, leading to diffusion-controlled termination.

Although photocrosslinking is widely used in 3D bioprinting, the conditions under which it is carried out—such as light source, intensity, exposure time, wavelength, and photoinitiator concentration—can vary greatly between labs. This variation makes it hard to compare results across different studies, even when the same bioink and photoinitiator are used.

Since most photocrosslinking relies on free radical generation, it’s important to understand how these radicals affect cells. Free radicals can interact with cell membranes, proteins, and DNA, potentially causing cell damage. In bioprinting, it’s especially important to know how cells respond to these conditions so that the shape fidelity and printing resolution are preserved while still keeping cells alive and functional. This section introduces the different photoinitiators and light sources commonly used for photocrosslinking bioinks in 3D bioprinting.

#### 3.3.2. Free-Radical Photoinitiators

**Figure 15:**
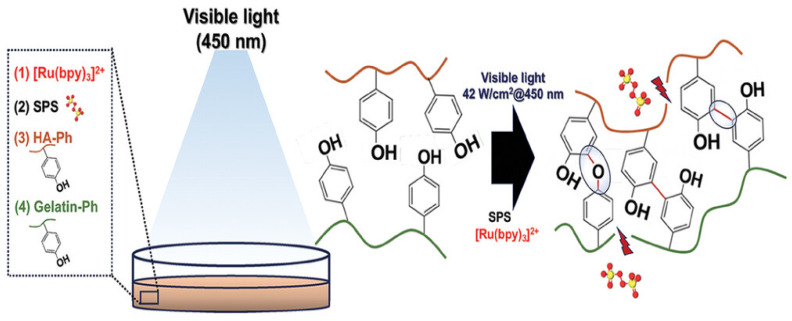
Schematic illustration of the contradictory impact of SPS on the formation of HA-Ph/Gelatin-Ph hydrogels and the degradation of the crosslinked polymer under visible-light irradiation (450 nm) in the presence of a photoinitiator, [Ru(bpy)_3_]^2+^ [162].

Free radical photoinitiators are the most commonly used type in bioprinting because their reaction pathways are well understood. These compounds absorb light and break down into free radicals, which then trigger the photocrosslinking process. Photoinitiators are generally classified into two types: Type I and Type II. Type I photoinitiators are single-component systems (such as irgacure and Lithium phenyl-2,4,6-trimethylbenzoylphosphinate (LAP)). They directly generate free radicals when exposed to light. Type II photoinitiators require two components: a photosensitizer (such as ruthenium) and a co-initiator (such as SPS). Together, they produce free radicals through a two-step light-activated process. In recent years, a new photoinitiating system has gained attention—tris(2,2-bipyridyl) dichlororuthenium (II) hexahydrate (Ru), a complex based on a transition metal. This compound is known for its strong ability to absorb visible light, with a molar extinction coefficient of 14,600 M^−1^cm^−1^ at 450 nm [158]. When exposed to visible light, Ru^2+^ is photoexcited and then oxidized to Ru^3+^ by donating an electron to a co-initiator, typically sodium persulfate (Figure 15). Once it accepts the electron, SPS breaks down into sulfate anions and sulfate radicals. These radicals can then kick off either free-radical chain polymerization or thiol–ene photo-crosslinking [159]. Interestingly, the photoexcited Ru^3+^ can also take part in redox reactions, especially with phenolic groups like tyrosine. When tyrosine derivatives are oxidized, it forms tyrosyl radicals, which can then pair up to create dityrosine bonds by reacting with nearby tyrosine groups. This adds another layer of functionality to Ru-based systems in biomaterial applications [160,161]

#### 3.3.3. Light Sources Used for Bioprinting and Light Attenuation

Photoinitiators work by absorbing light at a specific wavelength, so the light source must match the photoinitiator’s absorption range. One commonly used light source in bioprinting is the light-emitting diode (LED). LEDs offer several benefits, including low heat output, low energy use, affordable operating costs, minimal maintenance, portability, compact design, and safe, user-friendly operation. LEDs have a long history of use in areas like dentistry and 3D printing, including digital inkjet printing. Commercially, LEDs are available at several wavelengths—commonly 365, 385, 395, 405, 455, and 477 nm. In bioprinting, there’s a general trend: Type I photoinitiators usually absorb UV light, while Type II photoinitiators tend to absorb visible light. The intensity of the light and how well it matches the photoinitiator’s absorption range both play a key role in how fast and effectively gelation occurs.

An important factor to consider when designing a bioink or bioresin is how much it attenuates light, including the effects of all its components, such as rheology modifiers. When curing larger structures, light intensity can drop significantly over just a few millimeters. If the light becomes too weak, it may cause uneven reaction rates and crosslinking levels across the structure. This can result in a heterogeneous network, affecting the mechanical and biological performance of the printed 3D-construct.

To better understand and predict this behavior, researchers often use quantitative Beer–Lambert law calculations. These help estimate how much light is absorbed by the bioink over a certain distance. The Beer–Lambert law describes the drop in light intensity as it passes through a medium, and it can be expressed with the following equation:

$$I=I0\cdot e-\varepsilon cLI=I_{0}\cdot e^{-\varepsilon cL}$$

where:

I is the transmitted light intensity;
I0 is the incident light intensity;
ε is the molar extinction coefficient of the absorbing species;
c is the concentration of the absorbing species;
L is the path length (thickness of the material).

**Figure 16:**
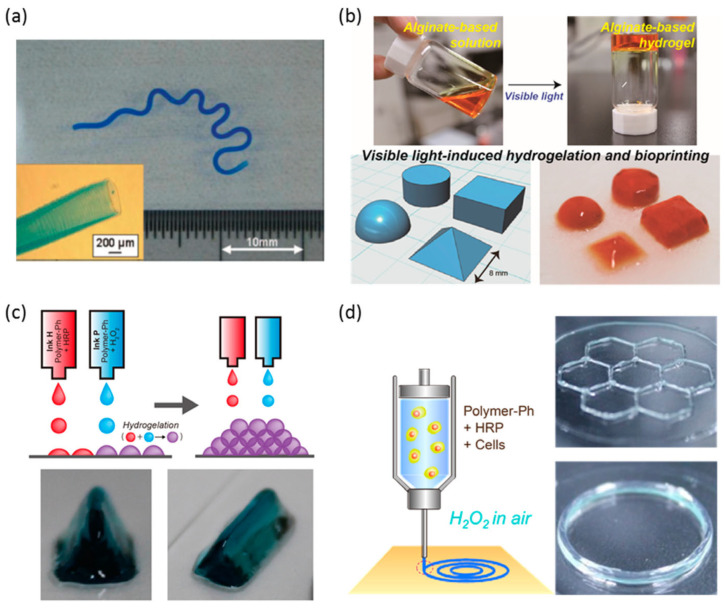
(**a**) Ca-alginate tube prepared through inkjet bioprinting, and hydrogel constructs obtained from (**b**,**c**) alginate derivative and (**d**) hyaluronic acid (HA) derivative possessing phenolic hydroxyl moieties through (**b**) stereolithography bioprinting, (**c**) inkjet bioprinting, and micro-extrusion bioprinting [22].

This equation helps in designing bioinks that allow for uniform light penetration and consistent crosslinking, especially in thicker or more complex structures [163]. The application scenarios of the three cross-linking methods mentioned above are illustrated as shown in Figure 16. In Figure 16, (a) shows an alginate and calcium mixture prepared by scaffold bioprinting, and (b) shows a scaffold prepared by photocrosslinking bioprinting. Figure 16c shows scaffolds prepared by bioprinting and microextrusion bioprinting. They are all derived from hydrogel structures obtained by (b) alginate gel and (d) phenolic hyaluronic acid with the HRP/H_2_O_2_ system.

Different modification methods have been employed to create biocompatible GG with high elasticity and improved mechanical properties. The most common derivative forms of gellan gum used for bioprinting are obtained as a result of methacrylation or acylation of the polymer, chemical grafting of peptides or copolymerization with other polymers such as poly(D,L-lactide-co-glycolid) (PLGA). Researchers have found that methacrylated gellan gum (GGMA) enabled UV-crosslinking and offered acceptable rheological properties. However, the resulting filament shape was unstable and led to low printing accuracy. To overcome this issue, calcium ions were suggested to pre-crosslink the polymer. Thus, the GGMA solution turned into a soft gel, which could then be UV-radiated. This two-step crosslinking technique was established as an effective method for using GGMA as a material for 3D printing [164]. The same methodology is also applicable for polyelectrolyte complex-based bioinks of methacrylated gellan gum with other polymers such as chitosan [165].

### 3.4. The Advantages of Anionic Polysaccharides Compared to Neutral Polysaccharides

The main advantage of anionic polysaccharides in 3D-printed tissue engineering, compared with neutral polysaccharides, comes from their negative charges. These charges include carboxyl and sulfate groups. These functional groups give the materials more flexible and biomimetic properties. This chemical difference makes them behave more like the natural extracellular matrix. As a result, they can better support cell environments, build stable structures, and promote tissue repair [22]. The advantages can be explained in several aspects:

First, anionic polysaccharides can form stable 3D structures through mild ionic cross-linking. Neutral polysaccharides such as agarose usually solidify only when temperature changes. In contrast, anionic polysaccharides can quickly form hydrogels at normal temperature and physiological conditions when divalent ions such as calcium ions are added. This cross-linking process is gentle. The process avoids damage to living cells from heat or ultraviolet light. This feature is very important for bio-3D printing. Researchers can also control the mechanical strength and stiffness of the scaffold by changing the ion concentration. Some anionic polysaccharides can combine ionic cross-linking with chemical cross-linking. This combination further improves mechanical strength and allows use in hard tissue repair, such as bone and cartilage. Second, anionic polysaccharides can mimic the extracellular matrix and support cell growth. The natural extracellular matrix in the human body contains many anionic polysaccharides, such as hyaluronic acid and chondroitin sulfate. Therefore, anionic polysaccharides already have biological advantages. Some materials, such as xanthan gum, have structures similar to glycosaminoglycans in the body. These materials provide cells with a familiar microenvironment. This environment supports cell adhesion, proliferation, and differentiation. The negative charges also attract water, which helps maintain a moist internal environment in the scaffold. At the same time, the negative charges can bind positively charged growth factors through electrostatic interaction. These growth factors include BMP and TGF-β. The scaffold can then release these factors slowly at the local site and guide cell differentiation. For example, studies show that scaffolds modified with carboxymethyl-κ-carrageenan can increase mineralization activity of pre-osteoblast cells and support bone formation [166]. Third, anionic polysaccharides provide many sites for chemical modification. The polymer chains contain many carboxyl and hydroxyl groups. These groups act as convenient positions for chemical reactions. Neutral polysaccharides usually have lower chemical activity and are harder to modify. For example, alginate does not naturally contain cell-binding sites. However, researchers can attach galactose molecules to alginate through its carboxyl groups. This modification improves the adhesion of hepatocyte cells such as HepG2 and helps them form functional cell spheroids. Researchers can also introduce antibacterial nanoparticles such as zinc oxide or polymers, such as chitosan, into the polysaccharide network. This strategy can give printed wound dressings antibacterial activity [165]. Fourth, many anionic polysaccharides have excellent rheological properties that improve printing accuracy. Materials such as xanthan gum and alginate often show shear-thinning behavior. The viscosity decreases when shear force is applied during extrusion through the printhead. This property makes the material easier to print. After extrusion, the shear force disappears, and the viscosity quickly increases again. The printed filaments can keep their shape and resist collapse. This behavior helps maintain high precision of the printed 3D structure. In summary, anionic polysaccharides provide mild ionic cross-linking, natural extracellular matrix-like behavior, and strong chemical modification potential. These materials are not only passive scaffolds in tissue engineering. These materials also act as active platforms that interact with cells and biological signals. Because of these properties, anionic polysaccharides show greater potential than neutral polysaccharides for building complex tissues and organs.

## 4. Application of Anionic Polysaccharides and Derivatives for 3D Bio-Printing in Tissue Engineering

Tissue engineering aims to create biomimetic structures that support cell attachment, proliferation, and differentiation. For this purpose, hydrogels based on anionic polysaccharides provide an excellent platform due to their softness, high water content, and similarity to natural extracellular matrices [40]. A wide range of synthetic and natural polymers—used either individually or in combination—have been developed into scaffolds for tissue regeneration [167]. Among them, alginate hydrogels are particularly notable for their biocompatibility, mild gelation, and ability to mimic the mechanical characteristics of native tissues [168].

**Figure 17:**
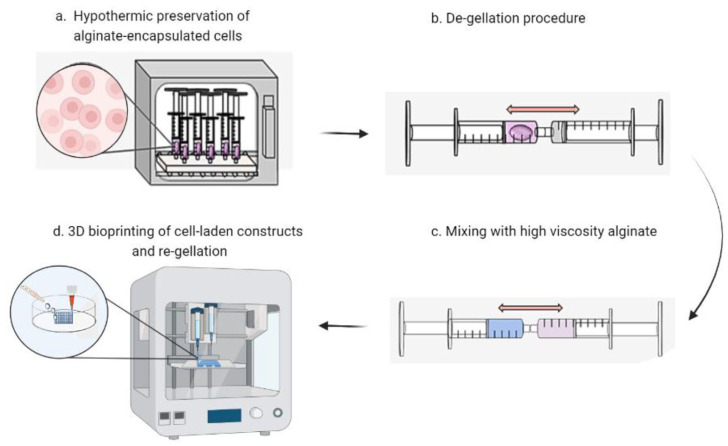
Schematic illustrating the process for printing stored cells. (**a**) Human adipose-derived stem cells encapsulated in BeadReady™ are stored for 1 week at controlled room temperature. (**b**) Alginate beads containing cells are dissolved using sodium citrate. (**c**) High viscosity alginate (HV-alginate) is mixed with the dissolved gel to increase ink viscosity. (**d**) Cell-laden bioink is extruded using a bioprinter and re-gelled using calcium chloride [170].

In practical applications, alginate hydrogels are often combined with other polymers, such as gelatin or chitosan, through physical or chemical crosslinking [169]. The carboxyl and hydroxyl groups on the alginate chain serve as reactive sites for forming stable crosslinked networks [18] (Figure 17a–d). Crosslinking with divalent cations like Ca^2+^ or enzymatic systems such as HRP/H_2_O_2_ allows precise control of gel strength, degradation, and biological response. Alginate hydrogels can encapsulate drugs or bioactive molecules, providing localized and sustained delivery for tissue regeneration. Compared with oral or injectable administration, hydrogel-based systems maintain a stable local drug concentration while reducing systemic side effects. Modified alginate formulations with tailored degradation rates and environmental responsiveness are being actively developed for next-generation drug delivery carriers.

**Figure 18:**
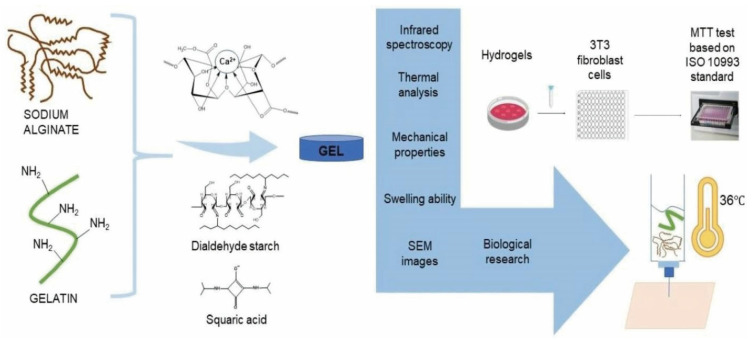
Gelatin-sodium alginate hydrogel is cross-linked with squaric acid and dialdehyde starch [172].

Studies have shown that a mixture of sodium alginate and gelatin can form stable hydrogels through ionic crosslinking with calcium chloride. When dialdehyde starch and squaric acid are used in this system, a blend of 2% sodium alginate and 6% gelatin shows good biocompatibility in a 1% CaCl_2_ solution. In addition, the covalent crosslinking created by dialdehyde starch and squaric acid improves the material’s printability. This makes the hydrogel suitable for 3D printing and strengthens its potential for use in tissue engineering (Figure 18) [171]**.**

**Figure 19:**
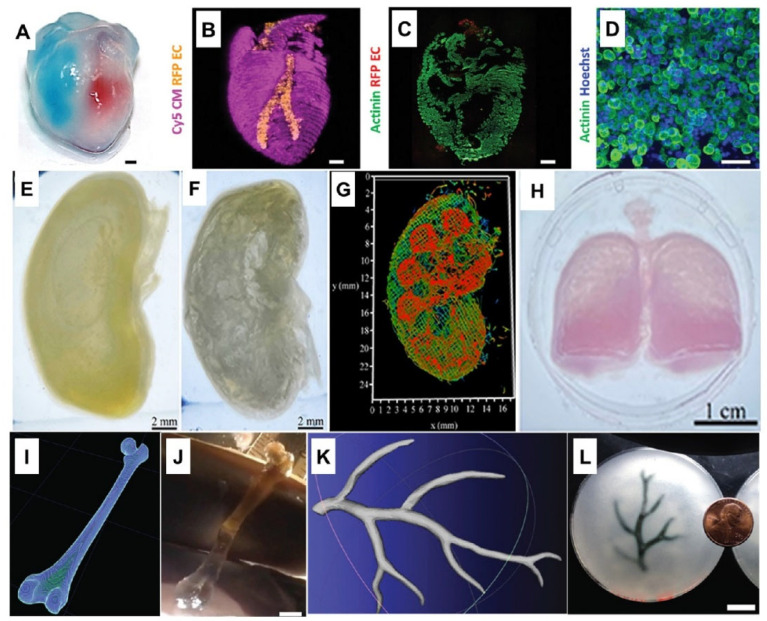
(**A**) 3D printing of thick and vascularized cardiac tissue, the hollow ventricles were distinguished with red and blue dyes. (**B**) Confocal images of a 3D bioprinted heart (pink highlights the cardiomyocytes and orange highlights the endothelial cells). (**C**,**D**) Cross-section images of immunostaining of sarcomeric actinin (green) [172]. Embedded 3D bioprinting of a complex kidney model using GelMA/sodium alginate bioinks. (**E**) Front view. (**F**) Rear view. (**G**) Confocal image showing renal vasculature and tubular system found in the natural kidney. (**H**) Photograph image of a 3D printed lung with trachea [175]. (**I**) Model of femur bone image procured from CT imaging. (**J**) The femur bone fabricated using the FRESH technique. (**K**) Model of human right coronary artery image procured from MRI imaging for FRESH printing. (**L**) The arterial tree was 3D printed using the FRESH technique using alginate ink and gelatin bath [174]. Scale bars: (**A**–**C**) = 1 mm, (**D**) = 50 μm (**E**,**F**) = 2 mm, (**H**) = 1 cm, (**J**) = 10 mm, (**L**) = 2.5 mm.

In 3D printing, polysaccharide-based hydrogels act as bioinks that can be printed into predefined shapes and structures. Commonly used anionic polysaccharides include alginate, hyaluronic acid, chondroitin sulfate, heparin, and polyglucuronic acid. These materials exhibit excellent printability, biocompatibility, and non-toxicity (Table 2). Noor et al. fabricated a customized patient-specific cardiac patch that is thick, vascularized, and perfusable, mimicking the anatomical features as shown in Figure 19A–D [172]. Zhang et al. utilized embedded printing using sub-microgel medium cationic-crosslinked κ-carrageenan to develop various complex structures like kidney, heart, lungs, and branched vasculature (Figure 19E–H) [173]. Hinton et al. printed femurs and hearts with soft hydrogels in thermo-reversible support baths, achieving excellent resolution and mechanical strength; a representative image is shown in Figure 19I–L [174].

The printing performance of a bioink depends not only on the material itself but also on the printing settings. The rheological properties of the bioink play a central role, but parameters such as pressure, speed, and nozzle size also strongly affect the final print [176]. Hydrogel precursors must have suitable viscoelastic properties before printing. These properties include shear thinning and yield stress behavior. Shear thinning means that the viscosity decreases when the shear rate increases. For example, the viscosity may decrease from about 10^4^ Pa·s to 100 Pa·s when the shear rate increases from 10^−2^ to 10^3^ rad/s. This behavior allows the material to flow easily through the nozzle during extrusion. At the same time, the material must show yield stress behavior. This property helps the printed filament keep its shape after extrusion. These rheological features are important for maintaining shape fidelity and controlling the final dimensions of the printed fibers during continuous printing. In extrusion-based bioprinting, the nozzle diameter usually ranges from 20 to 500 μm. The diameter of the printed hydrogel fibers is often slightly larger than the nozzle diameter. This increase occurs because of die swelling after the material exits the nozzle [177].

**Figure 20:**
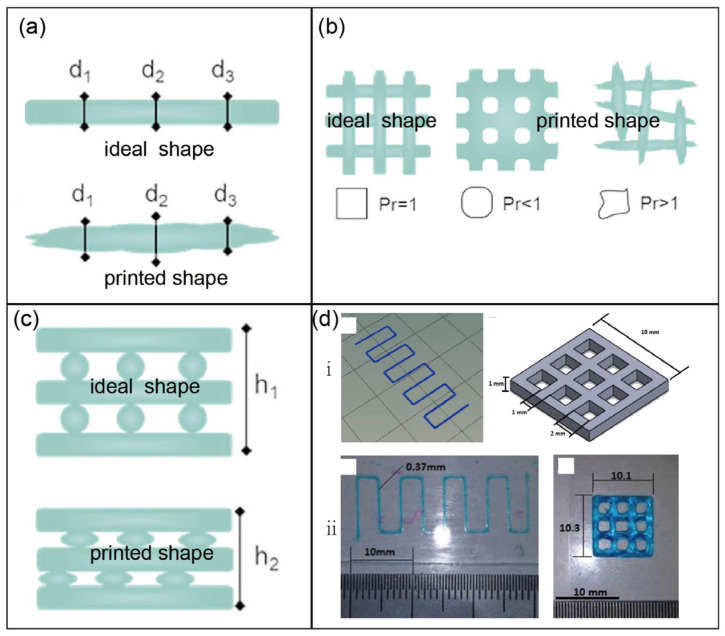
The assessment of shape fidelity: the assessment of cylindrical filament (**a**), pore (**b**), and height along the z-axis direction (**c**) (reprinted from Ref. [181]); (**d**) the differences between the design (**i**) and the printed constructs (**ii**) (reprinted with permission from Ref. [178], copyright 2018, Elsevier).

One simple way to evaluate print quality is to measure the width of the printed filament at certain points and compare it with the width defined in the design (Figure 20a). In recent years, semi-quantitative methods based on pore-shape fidelity have become more common. A dimensionless parameter, Pr (0 < Pr ≤ 1), is often used to describe how closely the printed pores match the designed pores (Figure 20b). Besides assessing shape accuracy in the x–y plane, it is also important to evaluate how much the structure collapses along the z-axis. A large difference between the designed height (h_1_) and the printed height (h_2_) indicates poor stability (Figure 20c). Several studies have described the typical mismatch between designed and printed structures and have illustrated these errors in detail [178] (Figure 20d). Extrusion bioprinting requires integrated control of material, process, and biological performance. Single parameters are insufficient. A unified framework should link rheology, print fidelity, and cell response to overall construct quality. Key rheological inputs include zero-shear viscosity and equilibrium modulus. Dynamic behavior is equally important. Shear-thinning, recovery after shear, and gelation rate govern extrusion and shape retention. These properties directly determine printability. Printed structures must be evaluated quantitatively. Relevant metrics include filament uniformity, pore regularity, and interlayer bonding. These indicators reflect structural fidelity and stability. Bio-inks show non-ideal behavior during printing. Wall slip, thixotropy, particle migration, and uneven crosslinking are common. These effects cannot be eliminated but must be controlled and quantified. Multi-objective optimization is essential. Printability metrics (e.g., shape fidelity) must be balanced with biological outcomes such as cell viability and matrix production. This enables rational formulation design [179]. Dynamic and reversible networks improve performance. Chemistries such as Schiff base and Diels–Alder reaction enable adaptable crosslinking. Supramolecular interactions further enhance self-recovery and tunability. These strategies decouple mechanical strength from cytocompatibility. Machine learning accelerates development. It predicts optimal compositions and printing conditions from complex datasets. This supports efficient navigation of multi-parameter design spaces [180].

**Table 2:** Application of anionic polysaccharides (APS) using 3D printing in tissue engineering.

APS	Other Composition	Property	Advantage	Application in Tissue Engineering	References
Alginate		Cancer recurrence and metastasis	Mitigating certain drawbacks inherent in conventional 2D cell culture	Crafting 3D scaffolds tailored to cultivate cancer stem cells (CSCs)	[182]
	Gelatin	Enriched with nanosilicates, substantial mechanical resilience	Capability to fabricate complete organs and tissues through printing techniques	Bone tissue engineering	[183]
	Gum tragacanth/nanohydroxyapatite	Remove ROS, improving cell viability, inducing osteogenesis and angiogenesis	Enhances its osteoinductivity	Bone tissue engineering	[184]
	CD8+ T cells	Antitumor		Cancer	[185]
Hyaluronic acid	REGRT, REG (a functional derivative of a red blood cell differentiation regulator)	Enhance acute excisional wound repair	Activating cellular migration	Wound healing	[186]
Xanthan gum		Enables the creation of both physical and chemical network structures	Elevated molecular weight	Drug delivery	[187]
		Reduces papain-induced osteoarthritis progression	Intra-articular injection	Bone tissue engineering	[188]
	Hydroxyethyl methacrylate-acrylic acid	Stable at 600 degrees	Super porous hydrogel (SPH)	Various tissues and biomedical fields	[189]
	Magnetic nanoparticles (MNPs)	Enhancing the bioadhesion and promoting neuronal differentiation of embryonic stem cells	Does not influence cell proliferation levels	Embryonic stem cells (ES-E14TG2a)	[190]
Carrageenan	Hydroxyapatite	Biocompatible and water-soluble		Bone tissue engineering	[191]
		Expression level of osteoblastin and the adsorption of protein	Increase viability	Bone tissue engineering	[80]
	Methylcellulose	High conductivity	Specific rheological attributes; high-resolution printing	Conductive scaffold	[192]
Pectin		Antibacterial	Controlled swelling	Wound dressings	[193]
	Chemically modifying	Releases drugs	Controllable	Drug delivery	[194]
Heparin and heparan sulfate	Cytokines	Promote osteogenesis	Combines the ability of various cytokines	Fracture healing	[195]
		Inhibiting tumor growth	Act on cell growth factor	Tumor treatment	[196]
Chondroitin sulfate		Promoting cell proliferation and adhesion	Inhibit the formation of biofilms	Wound healing	[197,198]
		Repair of nerve axons		Neural tissue engineering	[199]
	Other natural polysaccharides	Durability and mechanical support	Muscle regeneration	Muscle tissue engineering	[200]

**Figure 21:**
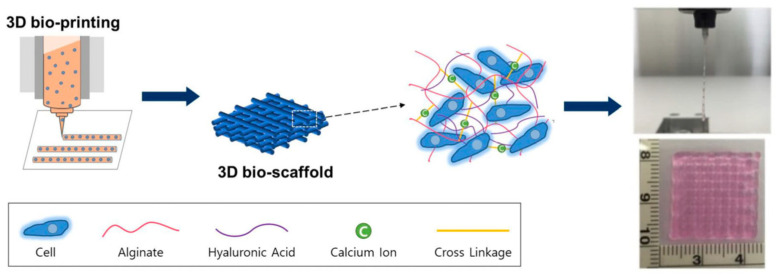
Bioink of hyaluronic acid and alginate for the printing of NIH 3T3 cells [201].

Hyaluronic acid has gained wide attention in tissue engineering because of its excellent biocompatibility. Lee and colleagues developed bioinks made from hyaluronic acid and sodium alginate mixed at different ratios (Figure 21). They found that the viscosity of the bioinks increased as the amount of hyaluronic acid increased. For example, the viscosities of S100H0, S90H10, and S70H30 were 883, 1211, and 1525 Pa·s, respectively. All of these bioinks could be printed into scaffolds using extrusion-based 3D printing. They also tested how NIH 3T3 cells behaved inside the printed scaffolds. After 7 days, the cells in all groups were alive and spread well throughout the structure. In addition, scaffolds with higher hyaluronic acid content supported stronger cell growth after 4 days. This shows that increasing hyaluronic acid can improve both printability and cell performance in the final construct [198].

**Figure 22:**
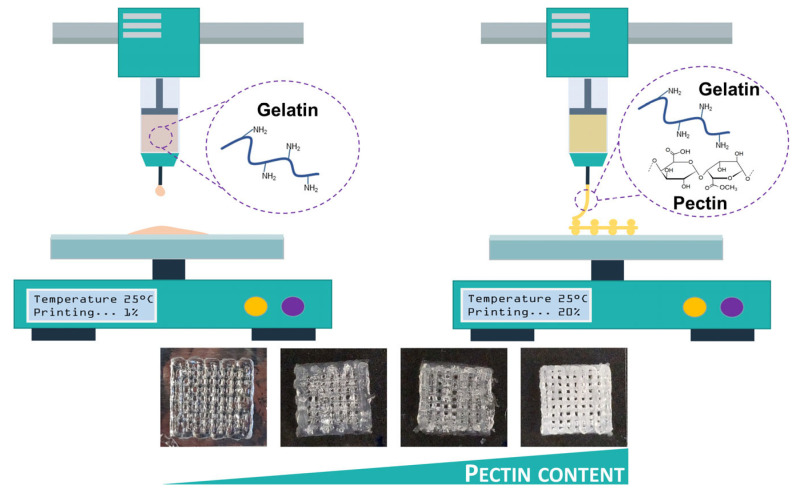
By combining extrusion printing with freeze-drying, a water-stable, three-dimensional, self-supporting gelatin-pectin-gptms scaffold with interconnected micro- and macro-pores was successfully obtained [204].

Pectin shows useful properties such as gelling, thickening, stabilizing emulsions, biocompatibility, and biodegradability. Because of these qualities, it is widely applied in both the food and pharmaceutical fields. Pectin can serve as a base material on its own or be combined with other additives (Figure 22). In this way, it is used to make pharmaceutical products such as gels, suppositories, films, and other forms that are important for skin tissue engineering [202]. Heparin and heparan sulfate are also widely used in tissue engineering because they take part in many biological activities, including blood lipid regulation, antiviral defense, and anti-allergic effects. Heparin has several protein-binding domains. It can bind with vascular endothelial growth factor (VEGF), fibroblast growth factor (FGF), and transforming growth factor-β (TGF-β) [203].

**Figure 23:**
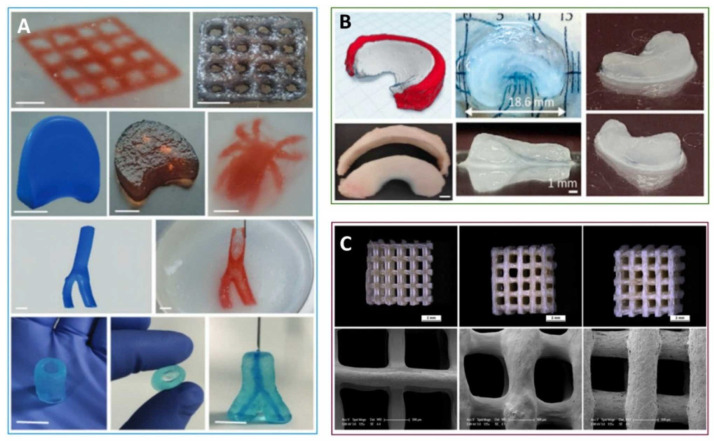
3D-printed structures using gellan gum-based bioinks: (**A**) intricate lattice; T7 intervertebral disc; intricate bulk structure in the form of a spider; carotid artery; tubular structure; scale bars = 10 mm [209]; (**B**) meniscus construct of 24 layers [210]; (**C**) light microscopic images of scaffolds (25 layers) (scale bar = 2 mm) and scanning electron microscopy (SEM) images of the scaffold surface (scale bar = 500 µm) [211].

Chondroitin sulfate has strong advantages, including biocompatibility, biodegradability, and very low toxicity. These features make it a valuable material for biomaterial development, especially in tissue engineering [205]. Among all traditional and advanced manufacturing methods, such as electrospinning, freeze-drying, solvent casting, and freeze–thawing, 3D printing is now the most widely used. It is especially important for making polysaccharide-based scaffolds for skin tissue repair. These methods also allow easy loading of drugs, growth factors, or bioactive nanomaterials into scaffolds. Such scaffolds can promote blood vessel growth, prevent infection, and reduce inflammation [206]. Gellan gum is a useful polymer in the development of bioinks that contain pro-angiogenic and anti-angiogenic endothelial cells. These bioinks can be supplemented with bioactive matrix-derived microfibers and can be printed into complex shapes(Figure 23A–C). A stent designed for wound healing should effectively prevent wound deterioration and possess a water absorption capacity ranging from 100% to 800%. This water absorption capability helps mitigate fluid accumulation, which could otherwise hinder the formation of new epidermal tissue [207]. This places specific demands on crosslinking agents and crosslinking methods. In practice, glutaraldehyde and EDC/NHS have been found to notably decrease the degradation rate of various carbohydrate polymers in both in vivo and in vitro aquatic environments [208].

**Figure 24:**
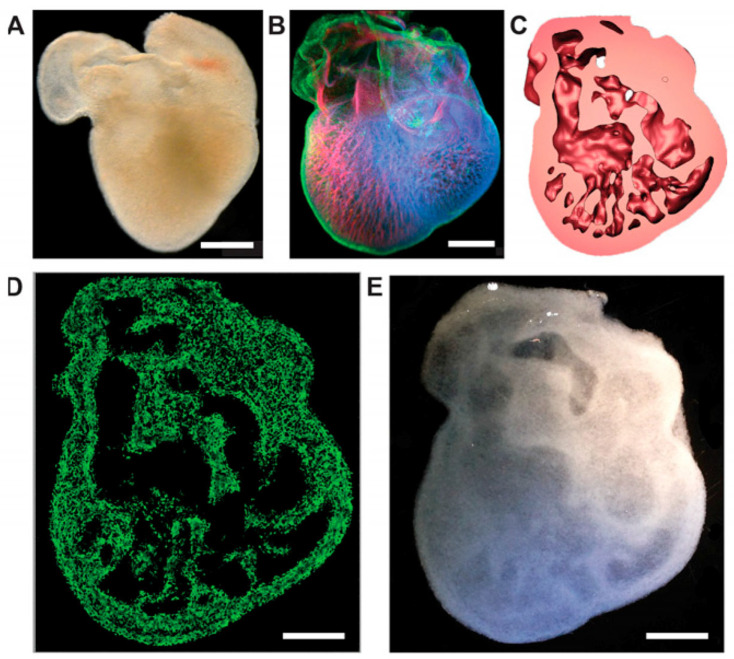
Method to bioprint a trabeculated embryonic heart using alginate-based bioinks. (**A**) Optical microscopy image of an embryonic chick heart; (**B**) a confocal microscopy 3D image of an embryonic chick heart stained for fibronectin (green), nuclei (blue), and F-actin (red); (**C**) a cross-section of the 3D model of the heart based on the confocal imaging data; (**D**) a cross-section of the 3D-printed heart in fluorescent alginate (green); (**E**) optical microscopy image of the bioprinted trabeculated embryonic heart. Figure modified from [215]. Scale bars, 1 mm (**A**,**B**) and 1 cm (**D**,**E**).

Alginate supports cell encapsulation during extrusion. Early studies showed stable 3D printing of endothelial cells. This confirms its basic suitability as a bio-ink [212]. Alginate provides higher mechanical strength than fibrin. It maintains structure during and after printing. However, it has weak cell interaction. Cells show limited adhesion, slow growth, and reduced differentiation. This limits tissue formation [213]. Alginate gels quickly after extrusion. This enables fast shape fixation and high structural stability. It is suitable for building large and complex constructs [214]. Alginate works well with support-bath printing. It maintains shape during the fabrication of intricate geometries. This allows printing of anatomically relevant structures from medical imaging data. Scientists printed structures such as a femur, a coronary artery, a human brain model, and a trabecular embryonic heart (Figure 24). Alginate is reliable for printability and shape control. Its main limitation is poor bioactivity. Bio-ink design often combines alginate with other materials to improve cell response while retaining mechanical stability [215].

**Figure 25:**
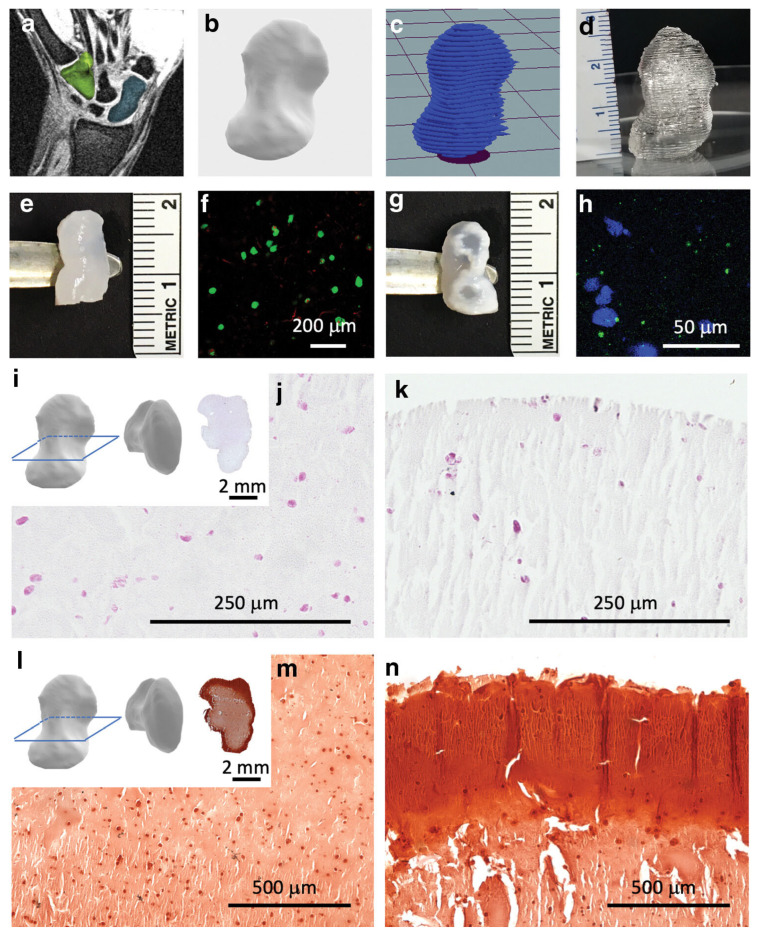
Bioprinting a scaphoid bone replacement. MRI images of the scaphoid of a healthy patient (**a**) (scaphoid in blue, hamate in green) were used to produce a 3D model (**b**), which was sliced (**c**) for 3D printing. (**d**) 3D printed full-size scaphoid printed with Pluronic. (**e**) Scaled down (50%) alg-CaCl2 printed scaphoid after 3D printing and crosslinking. (**f**) Live/dead imaging of MSCs encapsulated in 3D printed scaphoids 1 day postprinting. (**g**) Alg-CaCl2 3D printed scaphoid after 14 days of in vitro culture. (**h**) Osteocalcin immunostaining of the 3D printed scaphoids after 14 days of culture (cell nuclei in blue and osteocalcin in green). (**i**) H&E histological analysis of the cross section of osteogenically-induced 3D printed scaphoids at the center (**j**) and periphery (**k**) of the construct. (**l**) Alizarin red staining of 3D printed scaphoids at the center (**m**) and periphery (**n**) of the construct. Color images are available online [216].

Scaphoid fractures are common and clinically challenging. Severe cases require bone grafts. The bone has a complex and patient-specific shape. Researchers have proposed 3D printing as a clinical method to treat these fractures. To print cell-loaded scaphoid grafts, the authors first built a 3D model of the scaphoid. The authors used MRI scans from healthy patients to create the shape (Figure 25a,b). The authors then sliced the model (Figure 25c) and generated G-code for printing. The authors selected sodium alginate as the bio-ink to produce anatomically accurate scaphoid constructs that contained cells. The authors first tested the printing process using Pluronic to confirm printability (Figure 25d). After this step, the authors printed a smaller model at 50% scale. The smaller size was more suitable for in vitro culture. The authors used alginate–calcium chloride bio-ink that contained 10 × 10^6^ mesenchymal stem cells per milliliter (Figure 25e). The authors then cultured the printed constructs in osteogenic medium for 14 days. The cells remained viable one day after printing (Figure 25f). After 14 days in osteogenic medium, the printed scaphoid constructs showed formation of a calcified matrix (Figure 25g). The constructs also showed positive staining for osteocalcin (Figure 25h). Histological analysis of cross-sections showed that cells were present in both the center and the outer regions of the construct (Figure 25i–k). Alizarin red staining showed uneven mineral formation in the construct. The staining results showed higher calcium deposition at the outer regions compared with the center (Figure 25l–n).

These studies showed that alginate has strong mechanical properties and excellent printability. However, alginate still requires modification to improve cell biological performance for tissue engineering applications. In practical use, anionic polysaccharides are rarely used alone. Researchers often mix them with other materials to improve overall performance. Each material provides different advantages. The goal is to achieve good printability, stable structure, and strong biological function at the same time. Pure alginate that relies only on ionic cross-linking is usually soft. The material can also swell easily after absorbing water, which may cause deformation. For applications such as bone or cartilage repair that require load bearing, this mechanical strength is not sufficient. Neutral polysaccharides such as agarose or methylcellulose also play useful roles. These materials show good thermal gelation or shear-thinning behavior. They provide the initial viscosity needed before printing. They also prevent structural collapse immediately after extrusion. Researchers can use them as temporary support frameworks. Afterward, anionic polysaccharides such as alginate form ionic cross-links and create a permanent, stable structure.

**Figure 26:**
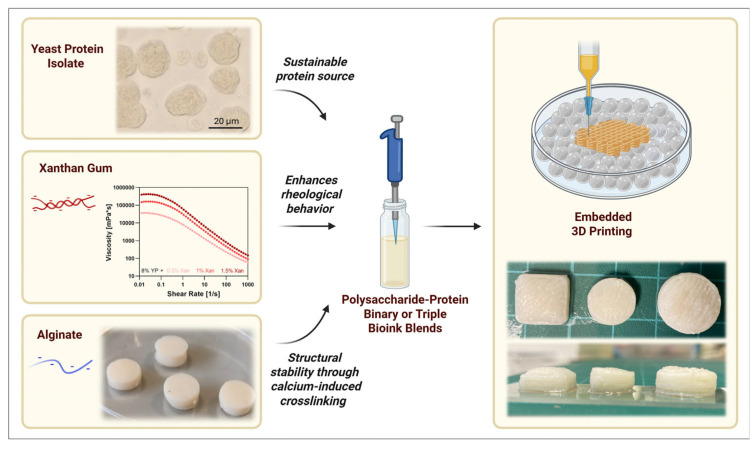
Illustration of Yeast Protein–Polysaccharide Bioink Blends for 3D Printing [217].

Researchers combine anionic polysaccharides with proteins or modified polymers. These components add cell adhesion sites and biological signals. The material becomes biologically active instead of passive. The main reason for material mixing is that human tissues are complex and layered. A single material cannot reproduce this complexity. Mixing materials allows complementary advantages and functional synergy. High concentrations of alginate (Alg) can reduce the thixotropic recovery ability of a material. This reduction means the material recovers its structure more slowly after shear stress stops. This behavior can negatively affect printing performance. Researchers developed a new bio-ink by combining yeast protein (YP), xanthan gum (XG), and alginate. This ternary system integrates the key advantages of each component. The YP–XG mixture provides elastic behavior and good thixotropic recovery. The Alg–XG combination contributes high viscosity and strong shear-thinning behavior. By combining these materials, researchers created a balanced bio-ink with improved overall performance [217]. In this formulation, alginate provides structural stability through calcium ion cross-linking. Xanthan gum contributes rheological flexibility and supports smooth extrusion. Yeast protein offers nutritional value and improves sustainability. This combination allows the material to function not only as a structural matrix but also as a biologically relevant system. Researchers successfully printed these bio-inks using embedded 3D printing technology. The printed structures maintained good shape fidelity after fabrication. After cross-linking, the triple-network hydrogel showed strong mechanical properties (Figure 26). The hydrogel maintained its volume after cross-linking and preserved structural integrity under compressive strain up to 70%. The material also recovered its shape after deformation. These results demonstrate high structural stability and mechanical resilience.

## 5. Conclusions

This review provides an overview of anionic polysaccharides and their applications in 3D bioprinting for tissue engineering. It summarizes the sources, extraction methods, molecular structures, and functional properties of eight major natural anionic polysaccharides—alginate, hyaluronic acid, xanthan gum, carrageenan, pectin, heparin, chondroitin sulfate, and polyglucuronic acid—as well as advances in synthetic analogs produced through chemical oxidation and modification. These polymers exhibit unique physicochemical and biological characteristics, including excellent biocompatibility, tunable gelation, and strong potential for chemical functionalization. Such traits make them ideal candidates for use as bioinks or hydrogel matrices in 3D bioprinting. Recent progress in crosslinking techniques, particularly HRP/H_2_O_2_ and ionic gelation methods, has expanded the design possibilities of polysaccharide-based hydrogels. Through these approaches, it is possible to engineer scaffolds with controlled mechanical strength, degradation rates, and bioactivity suited for various tissue regeneration applications. Overall, the synergy between anionic polysaccharides and 3D bioprinting technology presents significant opportunities for the creation of customized, cell-responsive, and biologically functional materials. These advances bring the field closer to the ultimate goal of fabricating complex, patient-specific tissues and organs for regenerative medicine.

## Figures and Tables

**Table 1:** Comparative overview of anionic polysaccharides for 3D bioprinting bio-inks.

Polysaccharide	Biological Properties	Mechanical Properties	Advantages	Limitations	Role in Bio-Inks	Typical Applications	Ref
**Alginate**	Non-toxic, biocompatible; biologically inert; poor intrinsic cell adhesion	Tunable stiffness via G/M ratio and Ca^2+^ concentration; moderate viscosity; weak under long-term hydration	Mild gelation, low cost, easy processing, widely available	Poor cell adhesion; limited bioactivity; mechanical weakening in aqueous environments	Structural matrix or carrier; often blended with gelatin, HA, cellulose, or nanofillers to improve bioactivity and strength	Cell encapsulation, cartilage, bone scaffolds, drug delivery	[18,19]
**Xanthan gum**	Cytocompatible; supports cell adhesion when modified or blended	Strong shear-thinning; high viscosity at low concentration; salt- and pH-resistant	Excellent rheology; stable under harsh conditions; industrial scalability	Limited bioactivity; weak gelation alone	Rheology modifier or printable ink after chemical modification (TEMPO oxidation, phenol grafting); enzyme or ion crosslinking	Soft tissue models, skin, cartilage, printable hydrogels	[41,91]
**Pectin**	Non-toxic; anti-inflammatory potential; protects cells from stress	Moderate stiffness; viscoelastic behavior similar to alginate blends	Plant-derived; biofriendly; inflammation reduction	Weak mechanics alone; limited printability	Functional additive to reduce immune response; blended with alginate or other matrices	Cell therapy, drug delivery, soft tissue scaffolds	[92,93]
**Hyaluronic acid (HA)**	Excellent biocompatibility and bioadhesion; actively regulates cell behavior	Poor mechanical stability unless modified; viscosity depends on MW	Native ECM component; promotes cell migration and proliferation	Fast degradation; weak shape retention	Bioactive component; blended or chemically modified (e.g., methacrylation, oxidation) to improve printability	Skin, cartilage, vascular and neural tissues	[94,95]
**Heparin**	Enhances growth factor stability; anticoagulant	Does not form gels alone; acts as functional additive	Improves BMP retention and activity	Non-specific binding; potential inhibition of key signaling pathways	Growth factor–binding additive; used at low doses or immobilized	Bone regeneration, growth factor delivery	[96,97]
**Gellan gum**	Cytocompatible; supports cell survival	Strong shear-thinning; improves filament stability	Excellent printability enhancer; good shape fidelity	Limited bioactivity; brittle at high concentration	Viscosity enhancer in GelMA-based inks; UV or ionic crosslinking	Cartilage, soft tissue scaffolds	[98]
**Carrageenan**	Generally biocompatible; cell survival depends on formulation	Thermo- and ion-responsive; weak mechanics under physiological conditions	Rapid gelation; tunable chemistry	Poor mechanical stability; requires modification	Methacrylation or blending with gelatin/alginate; UV + ionic dual crosslinking	Adipose tissue, soft tissue scaffolds	[90,99]
**Polyglucuronic acid (PGU)**	Excellent cytocompatibility; supports long-term cell viability	High viscosity; strong ionic and enzymatic gelation	Alginate alternative; strong printability; tunable chemistry	Limited commercial availability	Structural bio-ink; enzyme (HRP) or photo-crosslinking; blends with cellulose	Injectable gels, extrusion bioprinting, liver and soft tissue models	[100]
**Carboxymethyl cellulose (CMC)**	Biocompatible; low toxicity; limited intrinsic bioactivity	High viscosity; strong shear-thinning; good water retention	Excellent rheology control; low cost; abundant	Weak mechanical strength alone; slow degradation	Rheology modifier or composite matrix with alginate, gelatin, clay, or nanofibers	Wound dressings, cartilage, bone scaffolds, drug delivery	[101]

## Data Availability

The original contributions presented in this study are included in the article. Further inquiries can be directed to the corresponding author.

## Abbreviations

The following abbreviations are used in this manuscript:

3D	Three Dimensions
BMPs	Bone morphogenetic proteins
CMC	Carbox-ymethyl cellulose
ECM	Extracellular matrix
FGF	Fibroblast growth factor
G	α-L-guluronic acid
GAGs	Glycosaminoglycans
GEL	Gelatin no (3-glycidyloxypropyl)-trimethoxysilane
GELPect	Gelatin with Pectin
GG	Gellan gum
HA	Hyaluronic acid
HRP	Horseradish peroxidase
LAP	Lithium phenyl-2,4,6-trimethylbenzoylphosphinate
LED	light-emitting diode
SA	Sodium alginate
M	(1,4)-β-D-mannuronic acid
mPEG	methoxy polyethylene glycol
PGU	Polyglucuronic acid
SPS	sodium persulfate
TEMPO	2,2,6,6-tetramethylpiperidine-1-oxyl
TPP	Tripolyphosphate
VEGF	Vascular endothelial growth factor
XG	Xanthan gum
